# Supplementary material for: Curdepsidones B–G, Six Depsidones with Anti-Inflammatory Activities from the Marine-Derived Fungus Curvularia sp. IFB-Z10
Source: Mar Drugs. 2019 May 5;17(5):266. doi: 10.3390/md17050266 (PMC6562388; doi:10.3390/md17050266)
Supplement: Supplementary file 1 [file marinedrugs-17-00266-s001.pdf]

Curdepsidones B-G, Six Depsidones with Anti-inflammatory Activities from the Marine-Derived Fungus *Curvularia* sp. IFB-Z10

Yi Ding [1,†], Faliang An [1,†], Xiaojing Zhu[1], Haiyuan Yu[1], Liling Hao[1,*] and Yanhua Lu[1,*]

[1] State Key Laboratory of Bioreactor Engineering, East China University of Science and Technology, 130 Mei Long Road, Shanghai 200237, People's Republic of China

[*] Corresponding author: E-mail address: holiday_hao1988@126.com; Tel.: +86-2164253823 (L.H.); luyanhua@ecust.edu.cn (Y.L.); Tel +86-2164251185.

[†] These authors equally contribute to this work.

Supporting Information

Table of contents

Figure S1. ^{1}H NMR spectra (500 MHz, $CDCl_3$) of **1**....4
Figure S2. ^{13}C NMR spectra (125 MHz, $CDCl_3$) of **1**....5
Figure S3. HSQC spectra ($CDCl_3$) of **1**....5
Figure S4. HMBC spectra ($CDCl_3$) of **1**. 6
Figure S5. ROSEY spectra ($CDCl_3$) of **1**. 7
Figure S6. UV spectra (MeOH) of **1**. 7
Figure S7. ECD spectra (MeOH) of **1**....7
Figure S8. HRESIMS spectra of **1**....8
Figure S9. ^{1}H NMR spectra (500 MHz, $CDCl_3$) of **2**....8
Figure S10. ^{13}C NMR spectra (125 MHz, $CDCl_3$) of **2**. 9
Figure S11. HMQC spectra ($CDCl_3$) of **2**. 9
Figure S12. HMBC spectra ($CDCl_3$) of **2**. 10
Figure S13. UV spectra (MeOH) of **2**. 10
Figure S14. ECD spectra (MeOH) of **2**....11
Figure S15. HRESIMS spectra of **2**. 11
Figure S16. ^{1}H NMR spectra (500 MHz, $CDCl_3$) of **3**....12
Figure S17. ^{13}C NMR spectra (125 MHz, $CDCl_3$) of **3**. 12
Figure S18. HSQC spectra ($CDCl_3$) of **3**. 13
Figure S19. HMBC spectra ($CDCl_3$) of **3**. 13
Figure S20. UV spectra (MeOH) of **3**. 14
Figure S21. ECD spectra (MeOH) of **3**....14
Figure S22. HRESIMS spectra of **3**. 15
Figure S23. ^{1}H NMR spectra (500 MHz, $CDCl_3$) of **4**....15
Figure S24. ^{13}C NMR spectra (125 MHz, $CDCl_3$) of **4**. 16
Figure S25. HSQC spectra ($CDCl_3$) of **4**. 16
Figure S26. HMBC spectra ($CDCl_3$) of **4**. 17
Figure S27. UV spectra (MeOH) of **4**. 17
Figure S28. ECD spectra (MeOH) of **4**....18
Figure S29. HRESIMS spectra of **4**. 18
Figure S30. ^{1}H NMR spectra (500 MHz, CD_3OD) of **5**. 19
Figure S31. ^{13}C NMR spectra (125 MHz, CD_3OD) of **5**. 19
Figure S32. HSQC spectra (CD_3OD) of **5**. 20

Figure S33. HMBC spectra (CD_3OD) of **5**. 20
Figure S34. UV spectra (MeOH) of **5**. 21
Figure S35. ECD spectra (MeOH) of **5**........21
Figure S36. HRESIMS spectra of **5**. 22
Figure S37. ^1H NMR spectra (500 MHz, $CDCl_3$) of **6**........22
Figure S38. ^{13}C NMR spectra (125 MHz, $CDCl_3$) of **6**. 23
Figure S39. HSQC spectra ($CDCl_3$) of **6**. 23
Figure S40. HMBC spectra ($CDCl_3$) of **6**. 24
Figure S41. UV spectra (MeOH) of **6**. 24
Figure S42. ECD spectra (MeOH) of **6**........25
Figure S43. HRESIMS spectra of **6**. 25
Figure S44. Chemical structure of undetermined relative configurations of compounds **I**, **II** and **III** (enantiomers not shown). 26
Table S1. Energies of conformers at MMFF94x force field. 26
Table S2. Energies of compounds I, II and III at B3LYP/6-311G (d, p) in gas phase. 32
Table S3. Standard orientations of compounds I, II and III at B3LYP/6-311G (d, p) level in gas phase. 33
Table S4. Experimental (**1** and **2**) and computed (a and b) ^{13}C-NMR chemical shifts........51
Table S5. Experimental (**1** and **2**) and computed (a and b) ^1H-NMR chemical shifts........52
Table S6. Statistics of Ordinary Least Squares Linear Regression (OLS-LR) of experimental and computed ^{13}C- and ^1H-NMR chemical shifts. 53

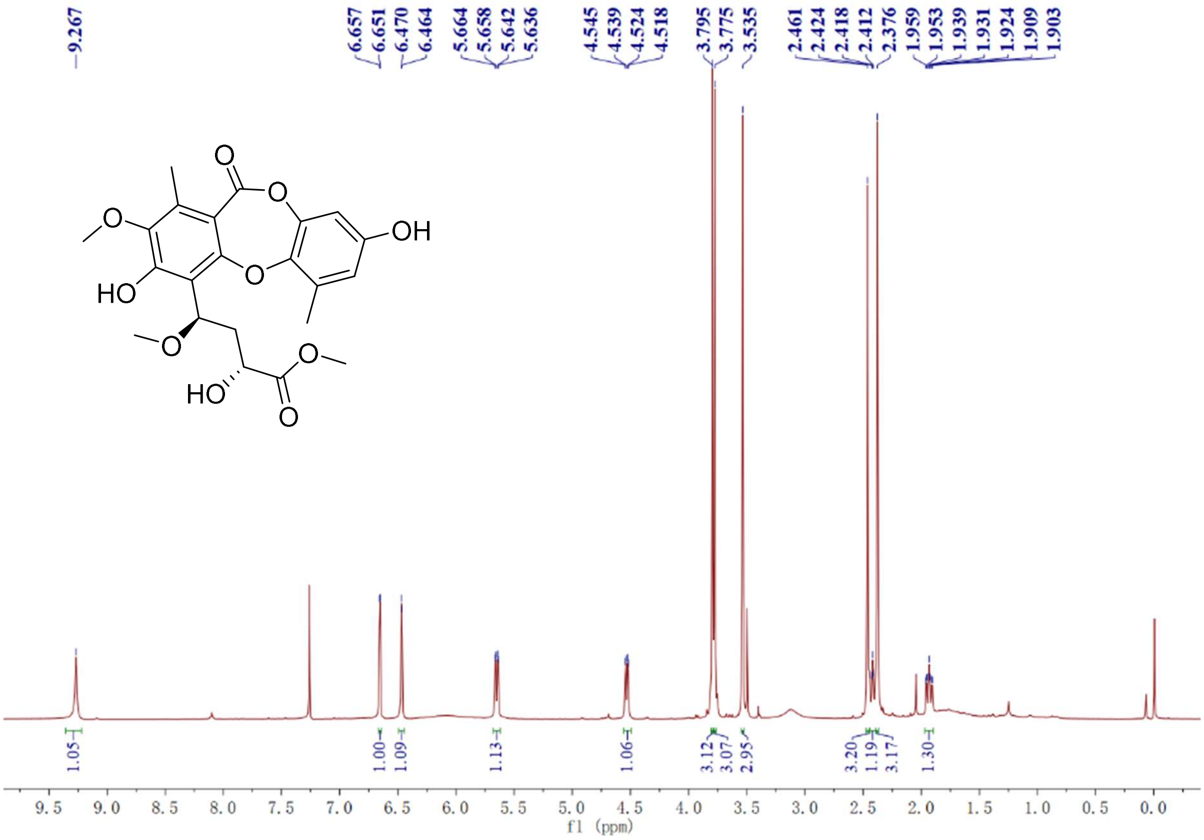

Figure S1. ^{1}H NMR spectra (500 MHz, $CDCl_3$) of **1**.

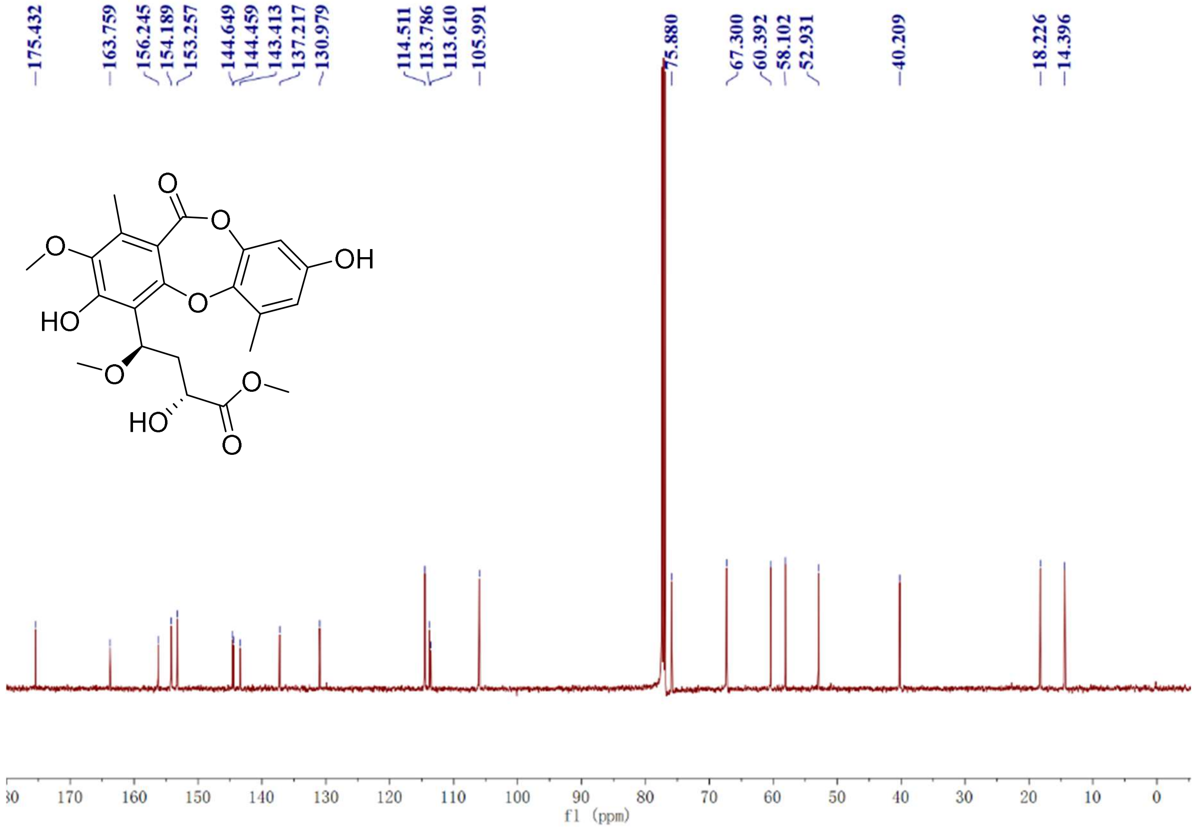

Figure S2. ^{13}C NMR spectra (125 MHz, $CDCl_3$) of **1**.

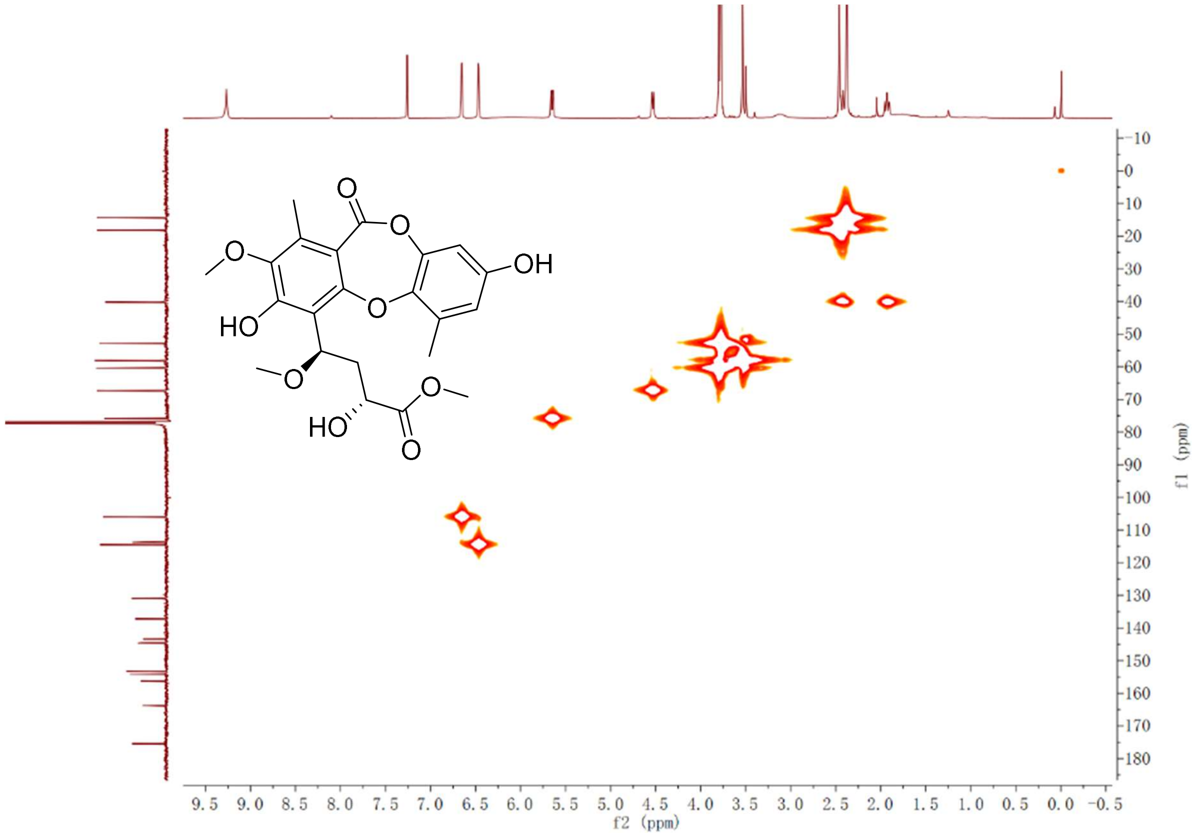

Figure S3. HSQC spectra ($CDCl_3$) of **1**.

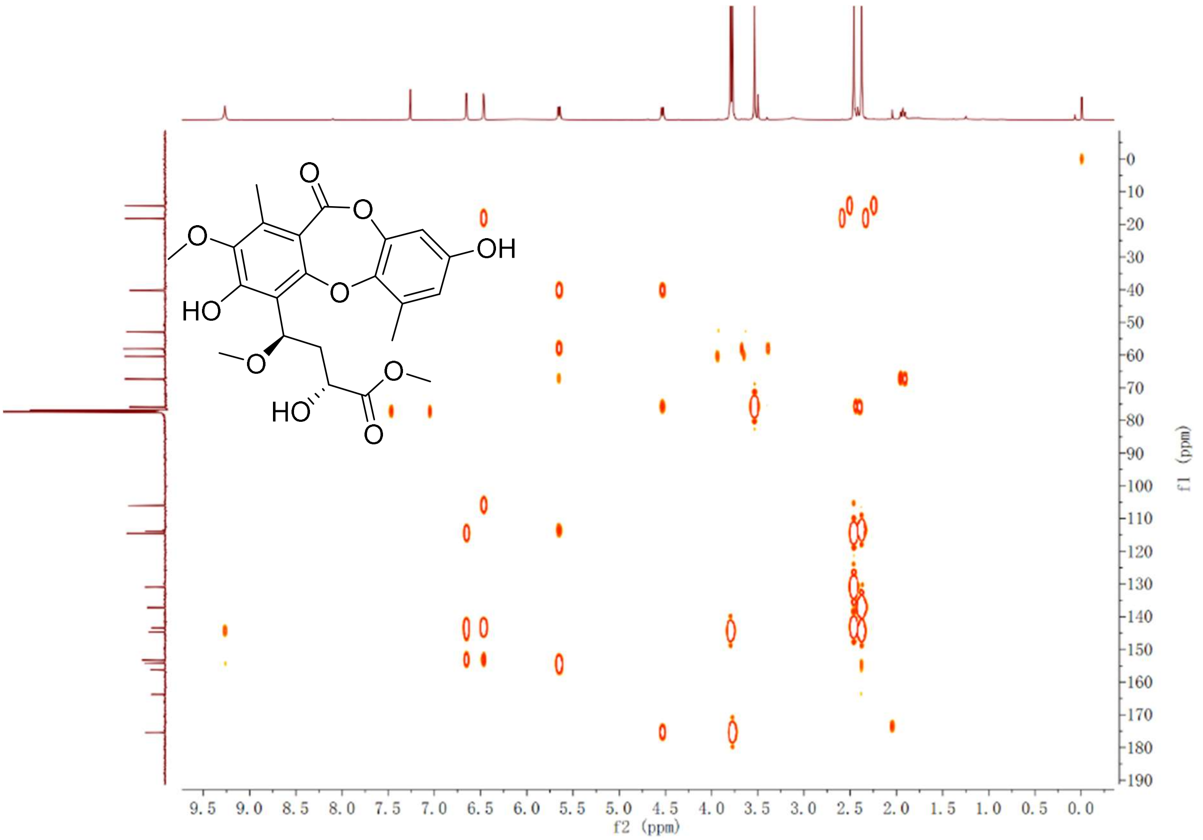

Figure S4. HMBC spectra ($CDCl_3$) of **1**.

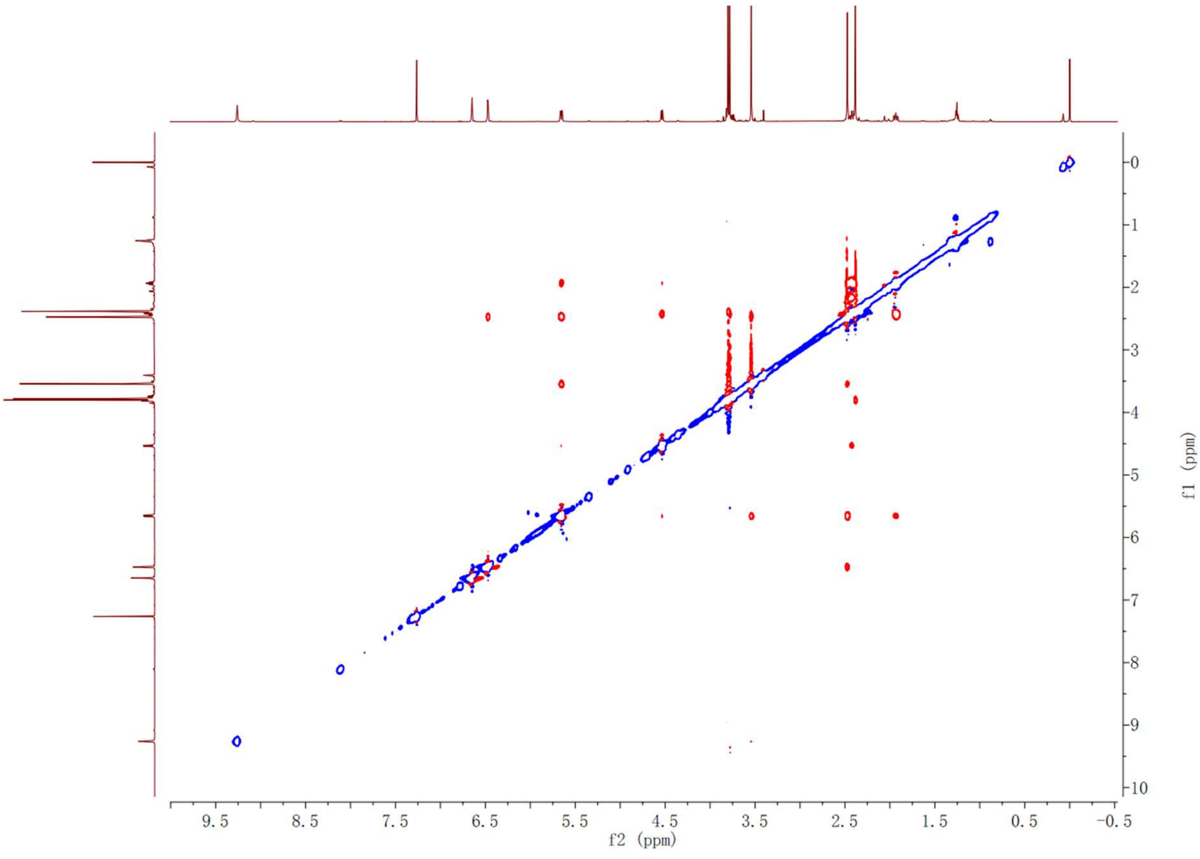

Figure S5. ROSEY spectra ($CDCl_3$) of **1**.

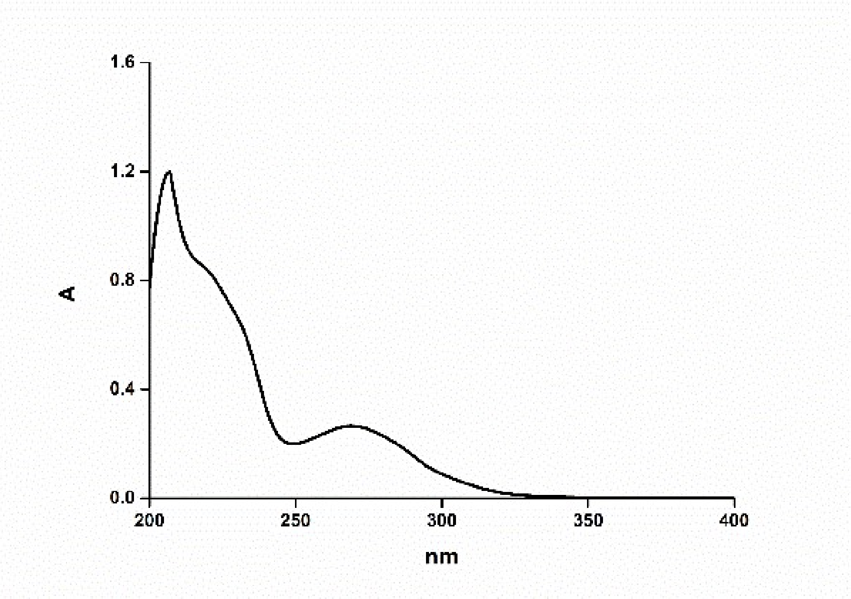

Figure S6. UV spectra (MeOH) of **1**.

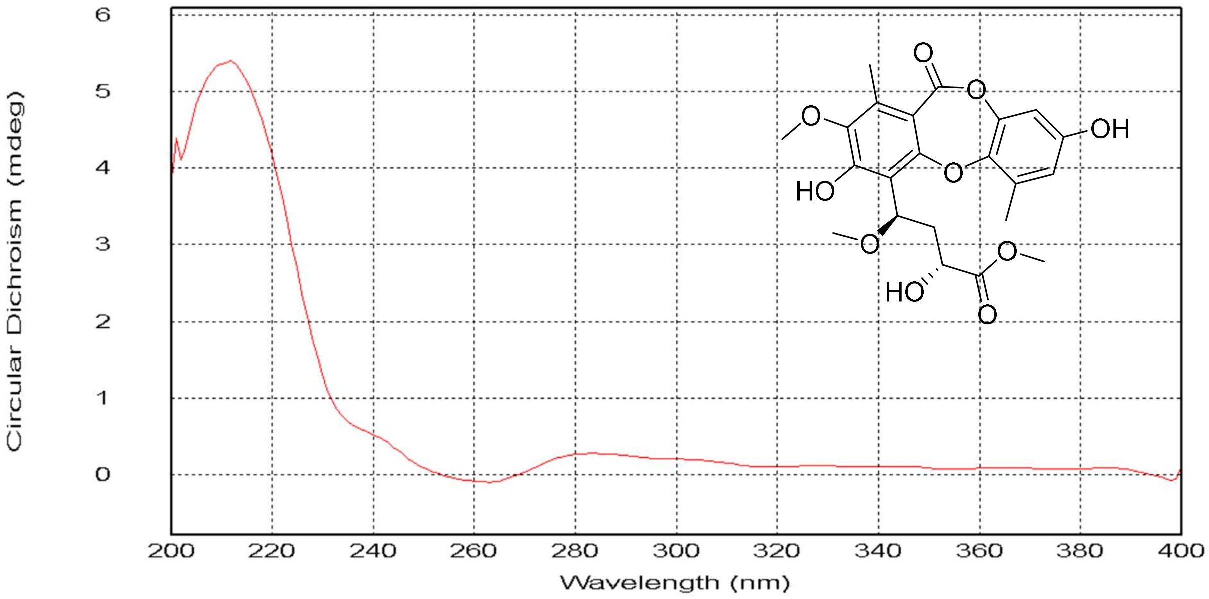

Figure S7. ECD spectra (MeOH) of **1**.

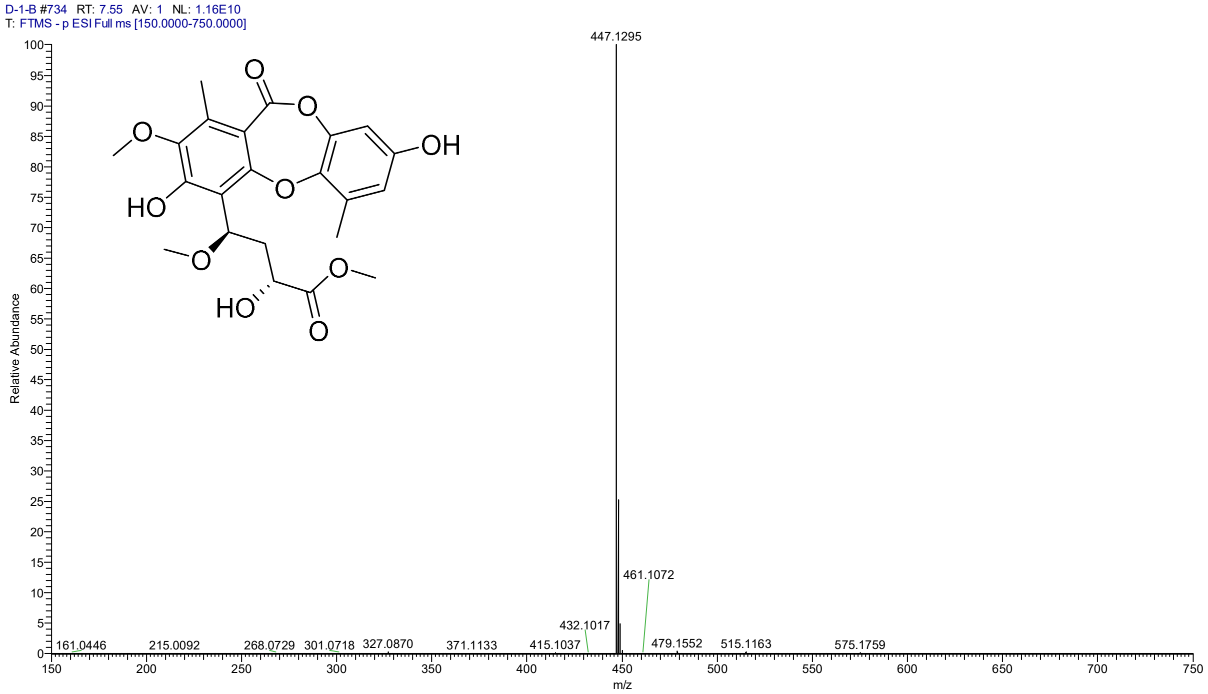

Figure S8. HRESIMS spectra of **1**.

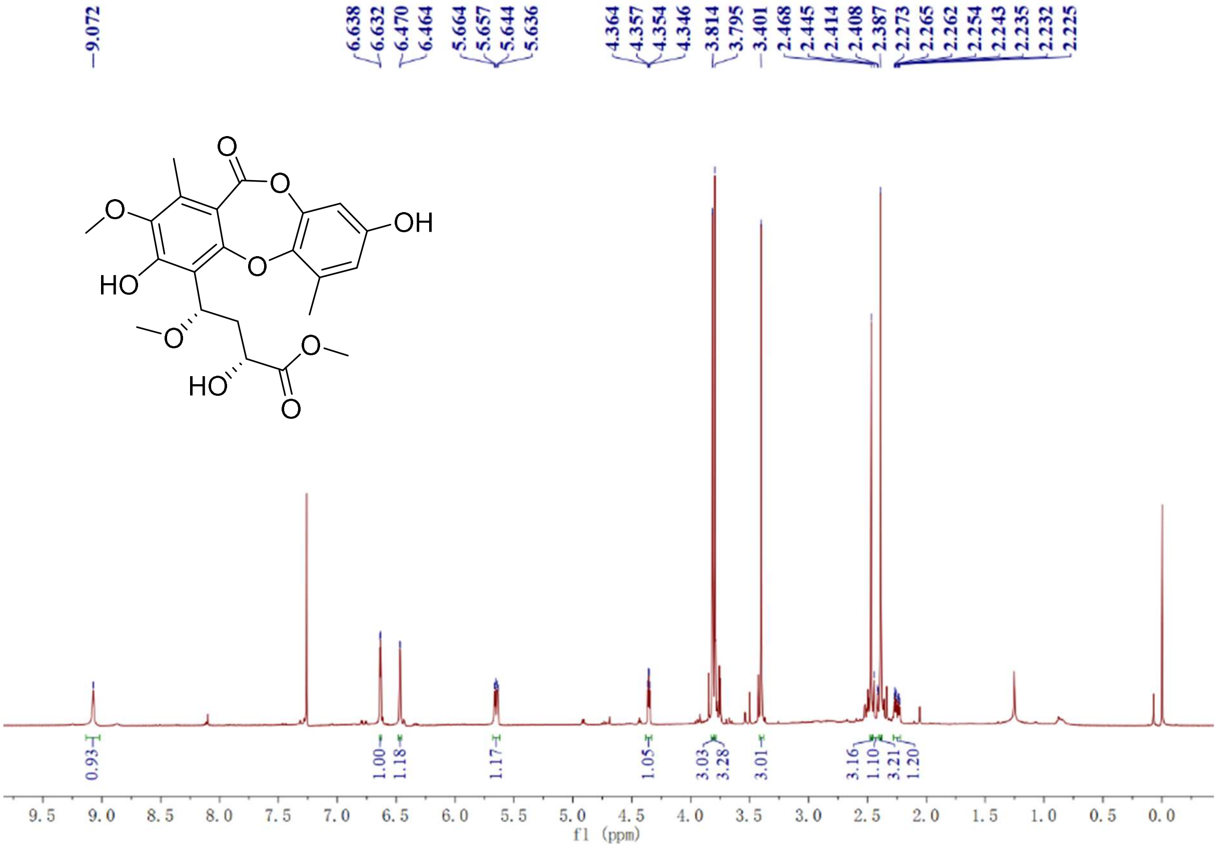

Figure S9. ^{1}H NMR spectra (500 MHz, $CDCl_3$) of **2**.

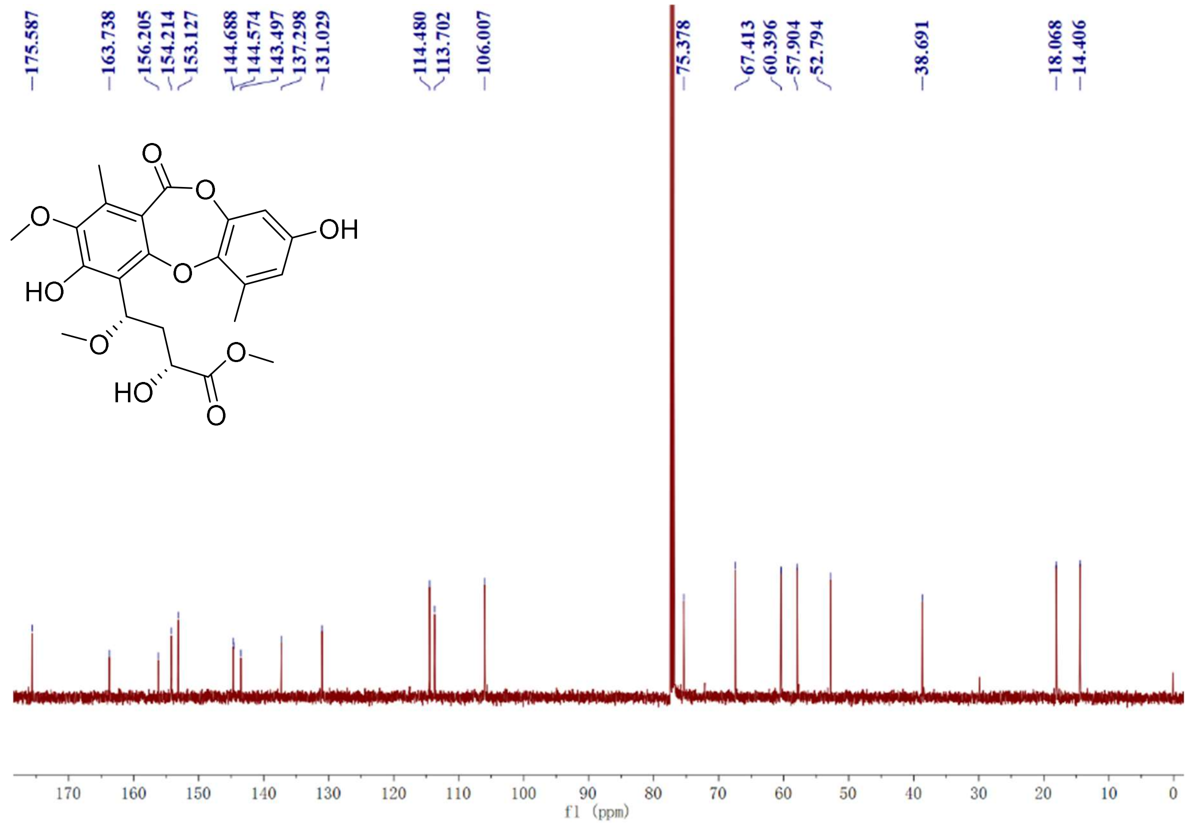

Figure S10. ^{13}C NMR spectra (125 MHz, $CDCl_3$) of **2**.

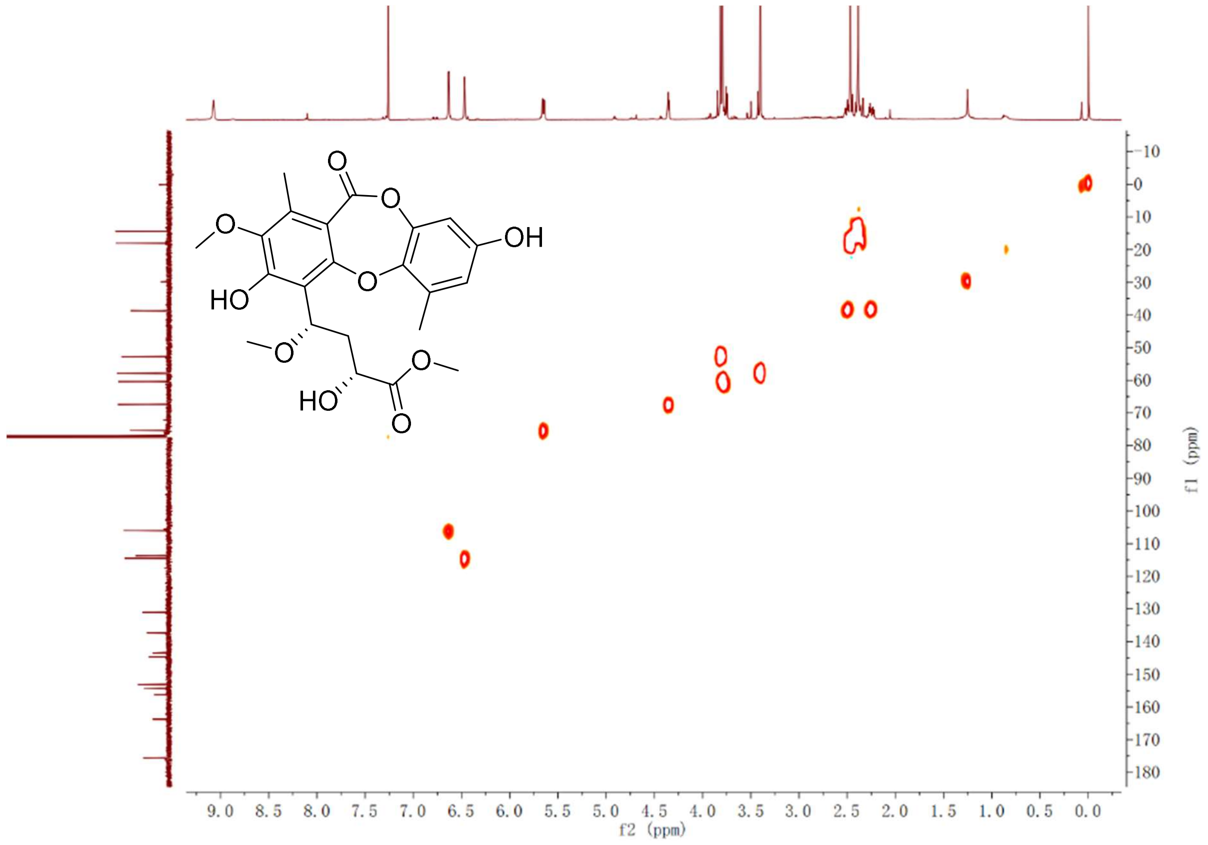

Figure S11. HMQC spectra ($CDCl_3$) of **2**.

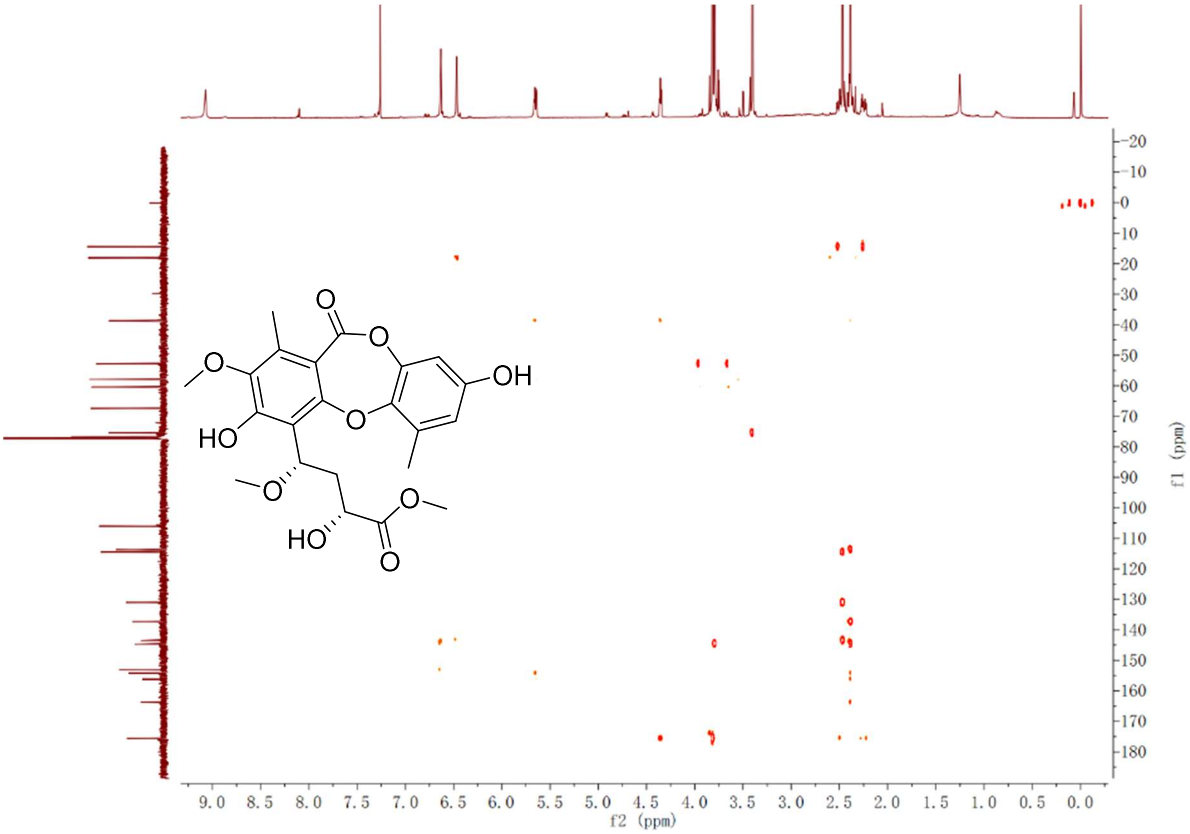

Figure S12. HMBC spectra (CDCl$_3$) of **2**.

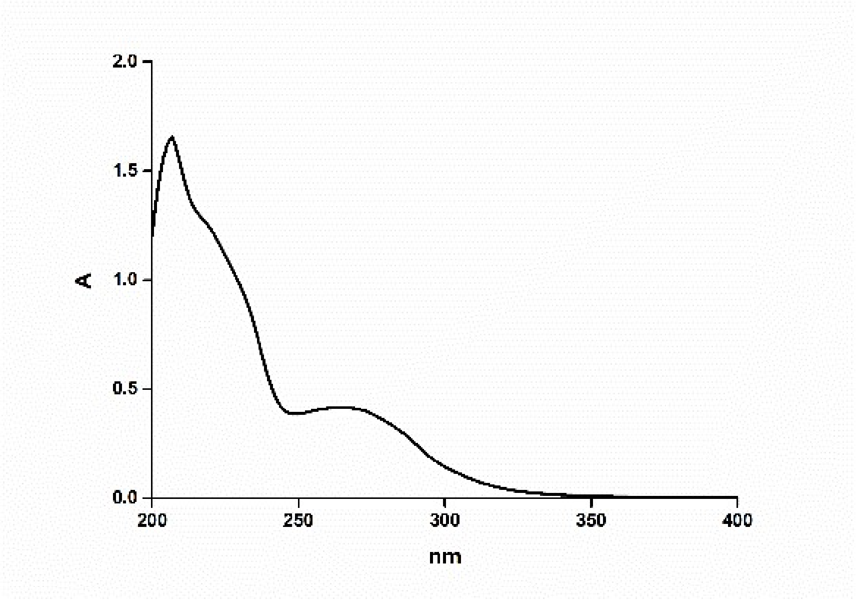

Figure S13. UV spectra (MeOH) of **2**.

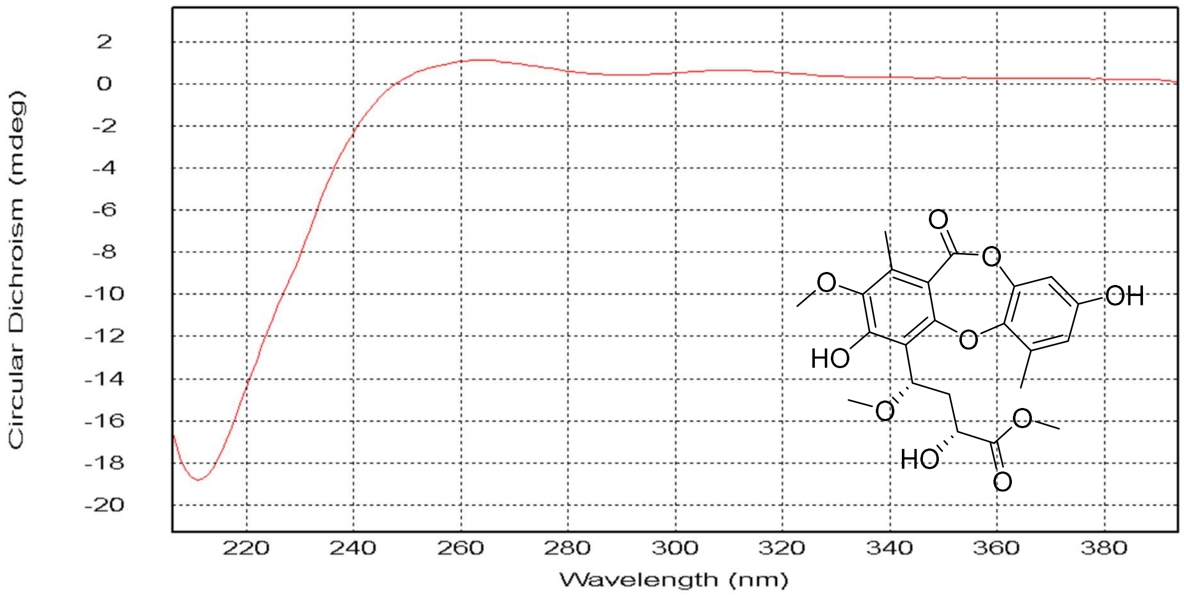

Figure S14. ECD spectra (MeOH) of **2**.

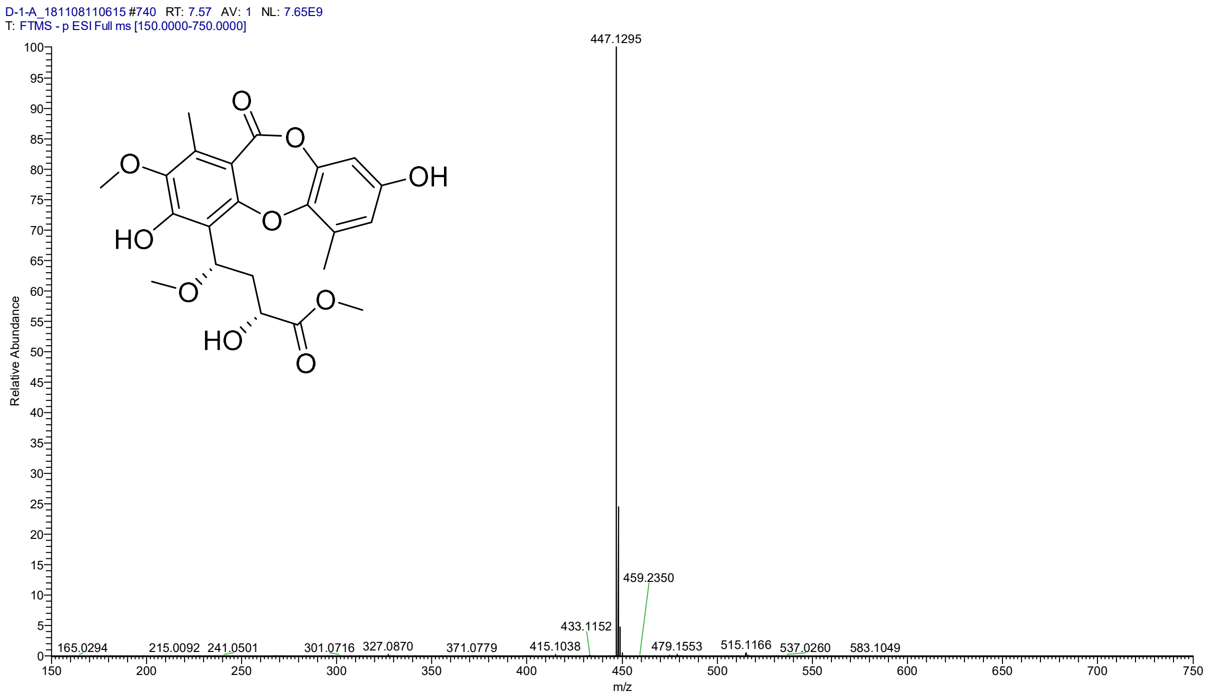

Figure S15. HRESIMS spectra of **2**.

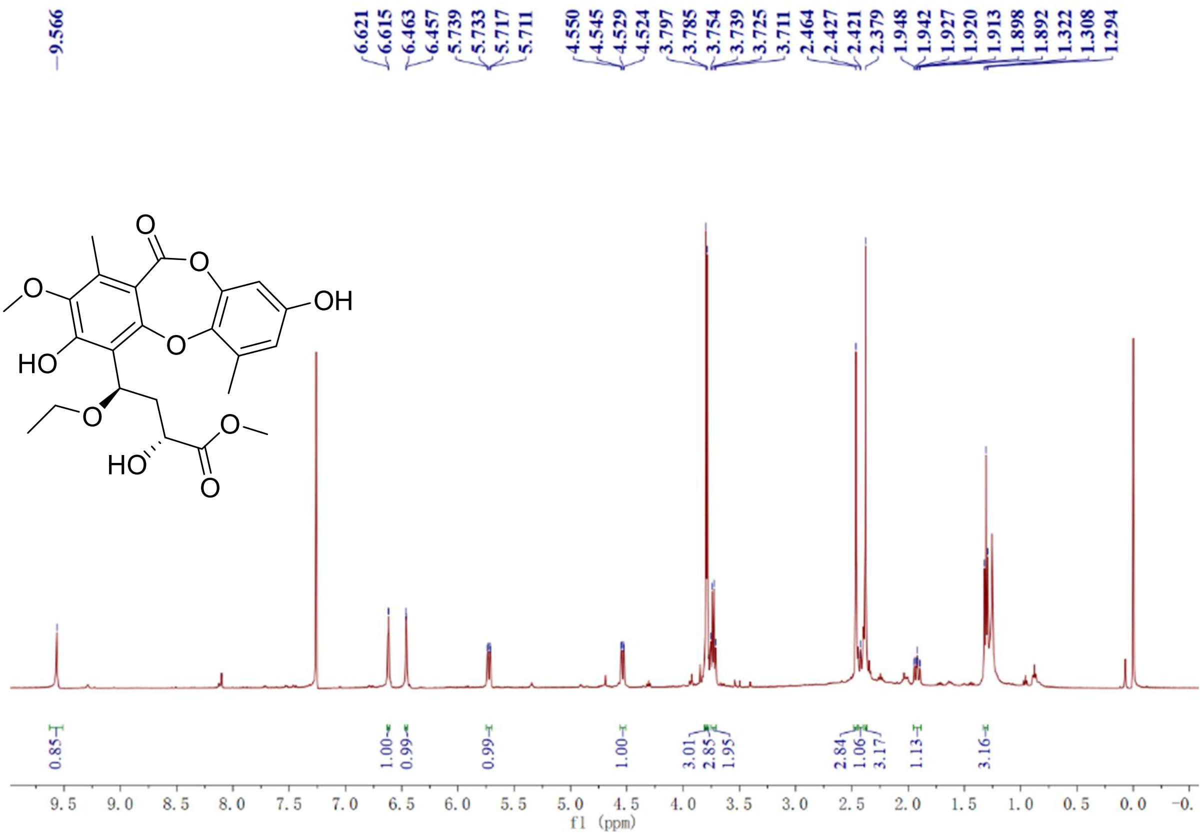

Figure S16. ^{1}H NMR spectra (500 MHz, $CDCl_3$) of **3**.

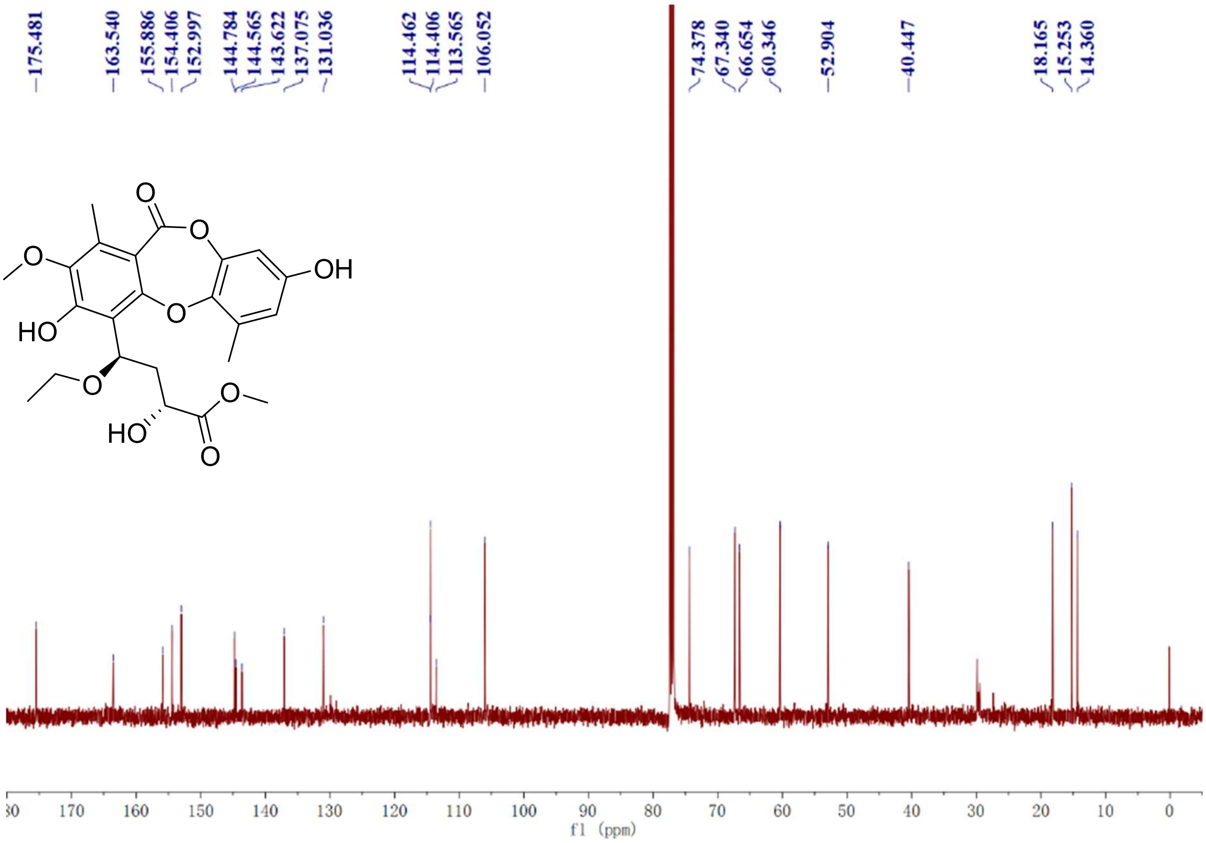

Figure S17. ^{13}C NMR spectra (125 MHz, $CDCl_3$) of **3**.

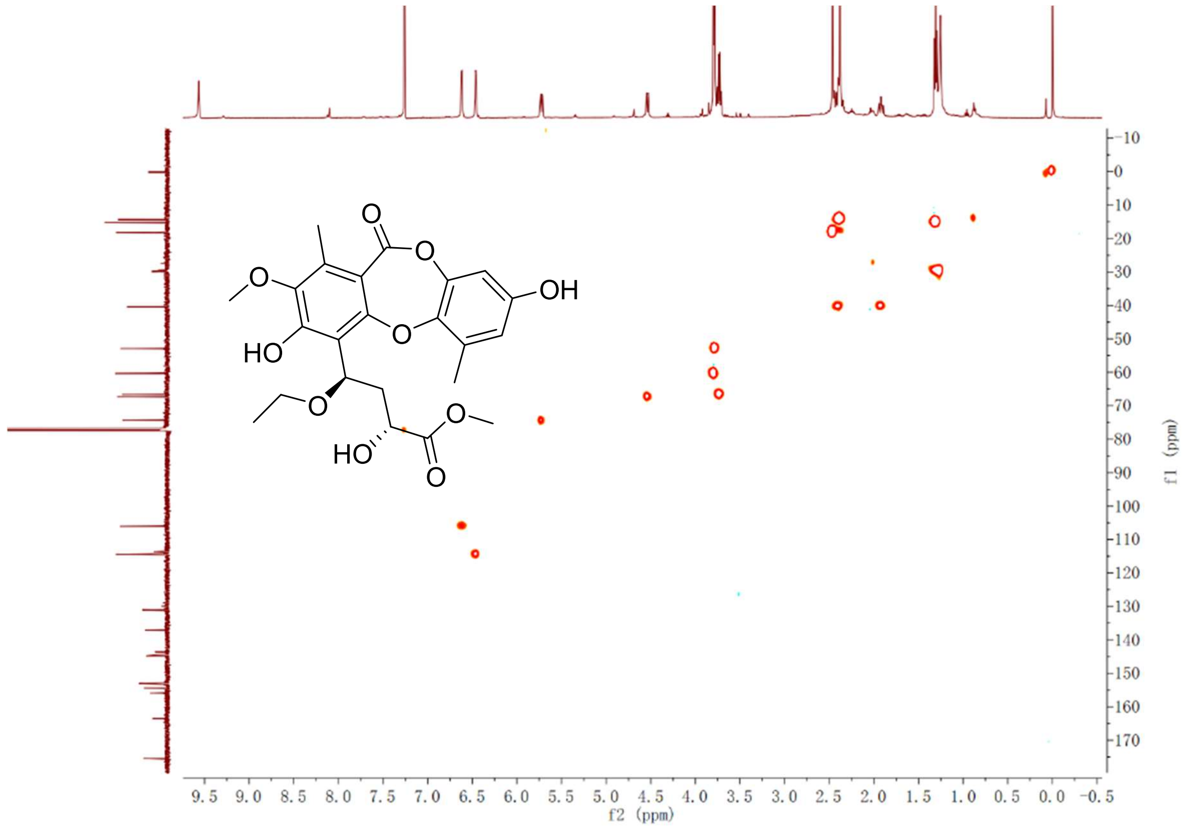

Figure S18. HSQC spectra ($CDCl_3$) of **3**.

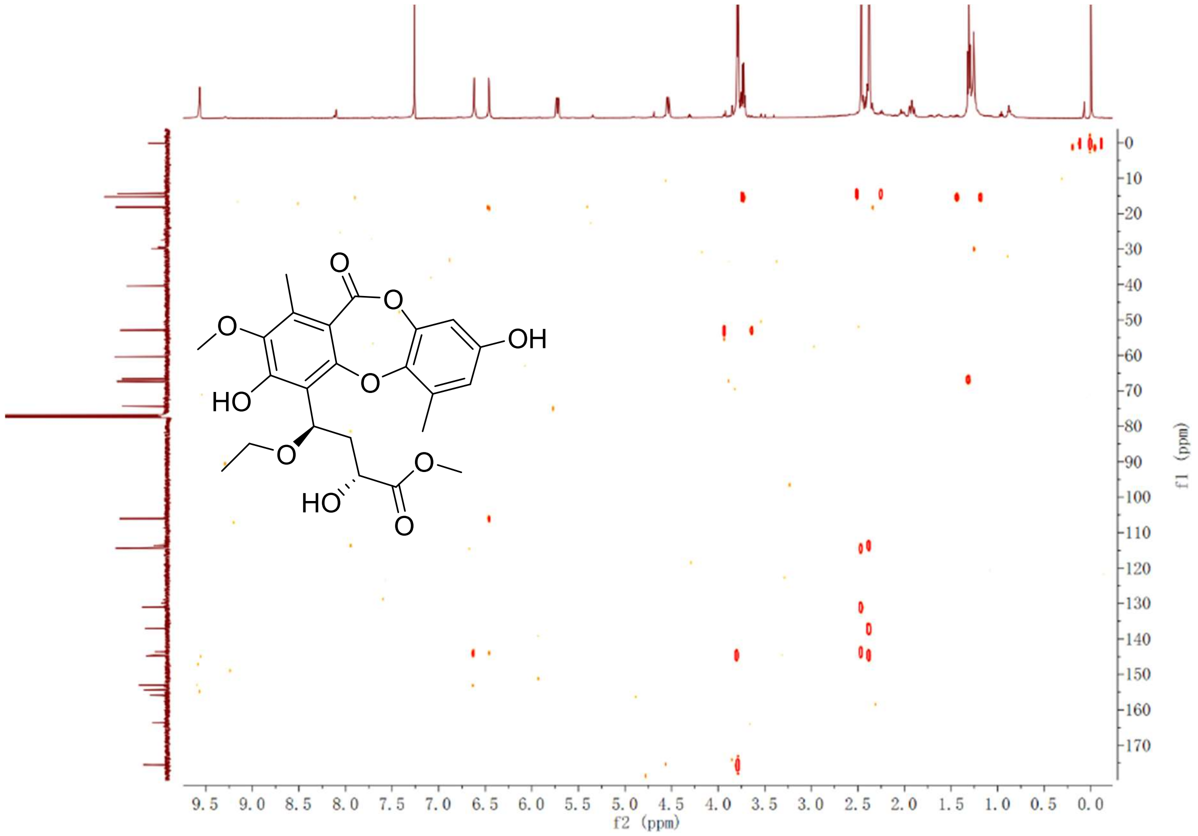

Figure S19. HMBC spectra ($CDCl_3$) of **3**.

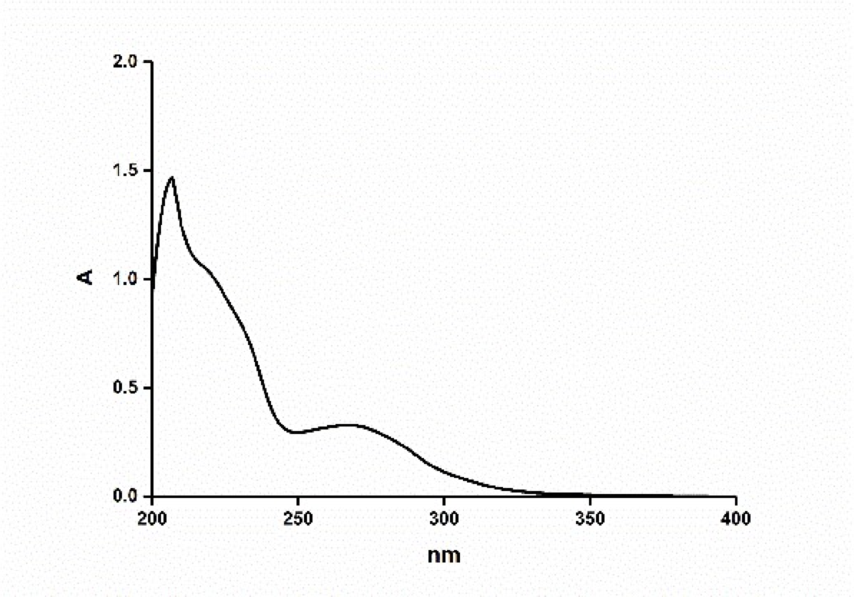

Figure S20. UV spectra (MeOH) of **3**.

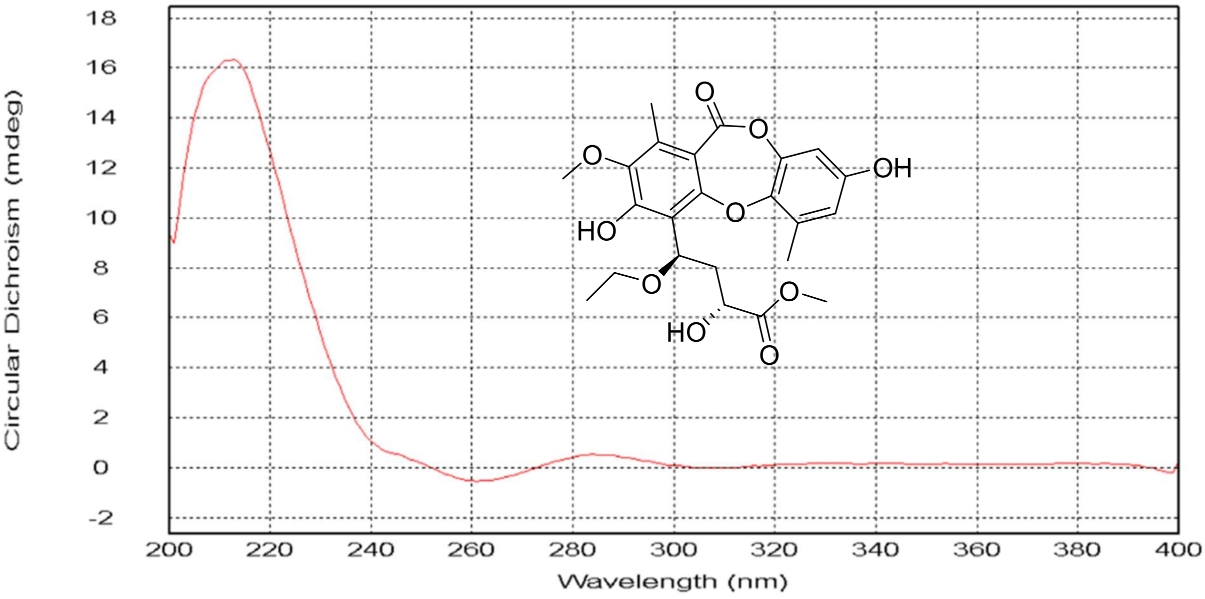

Figure S21. ECD spectra (MeOH) of **3**.

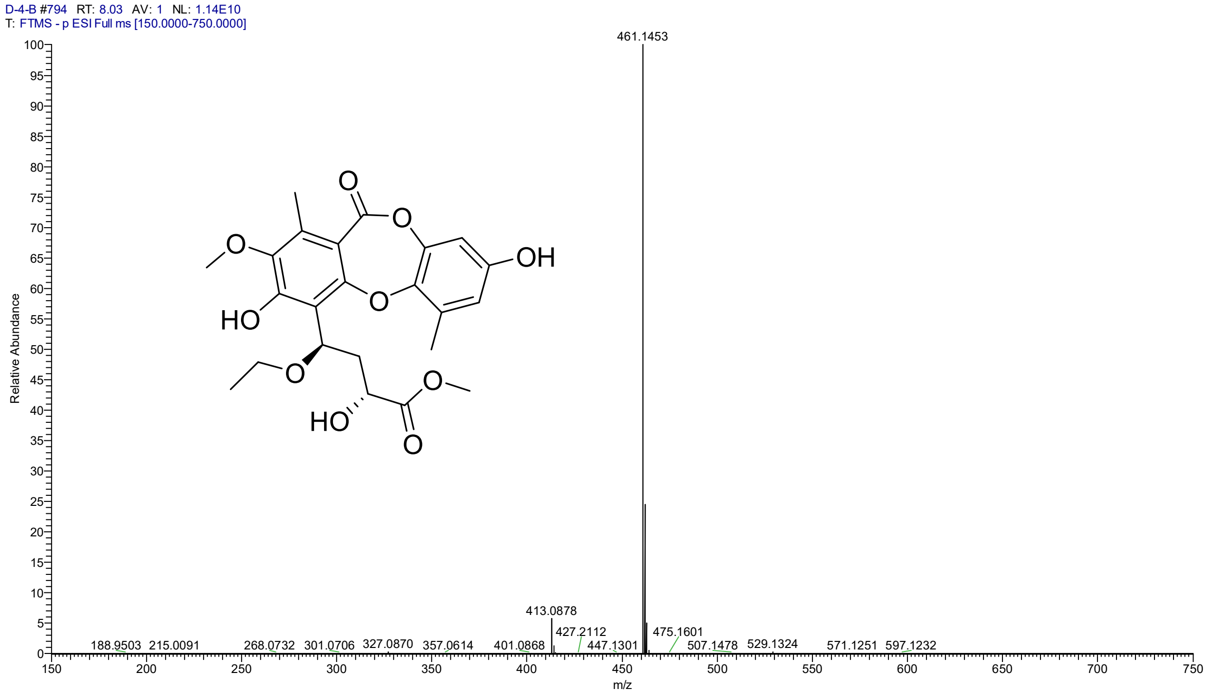

Figure S22. HRESIMS spectra of **3**.

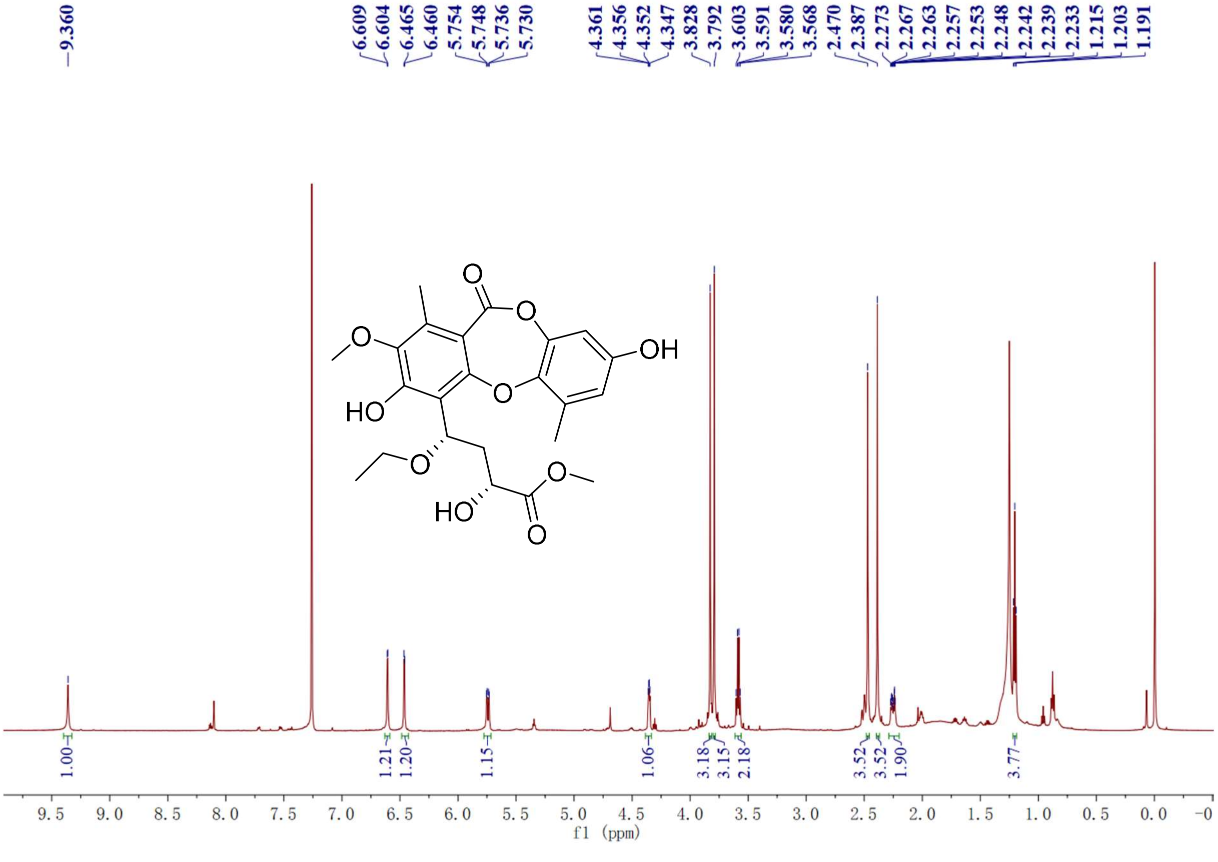

Figure S23. ^{1}H NMR spectra (500 MHz, $CDCl_3$) of **4**.

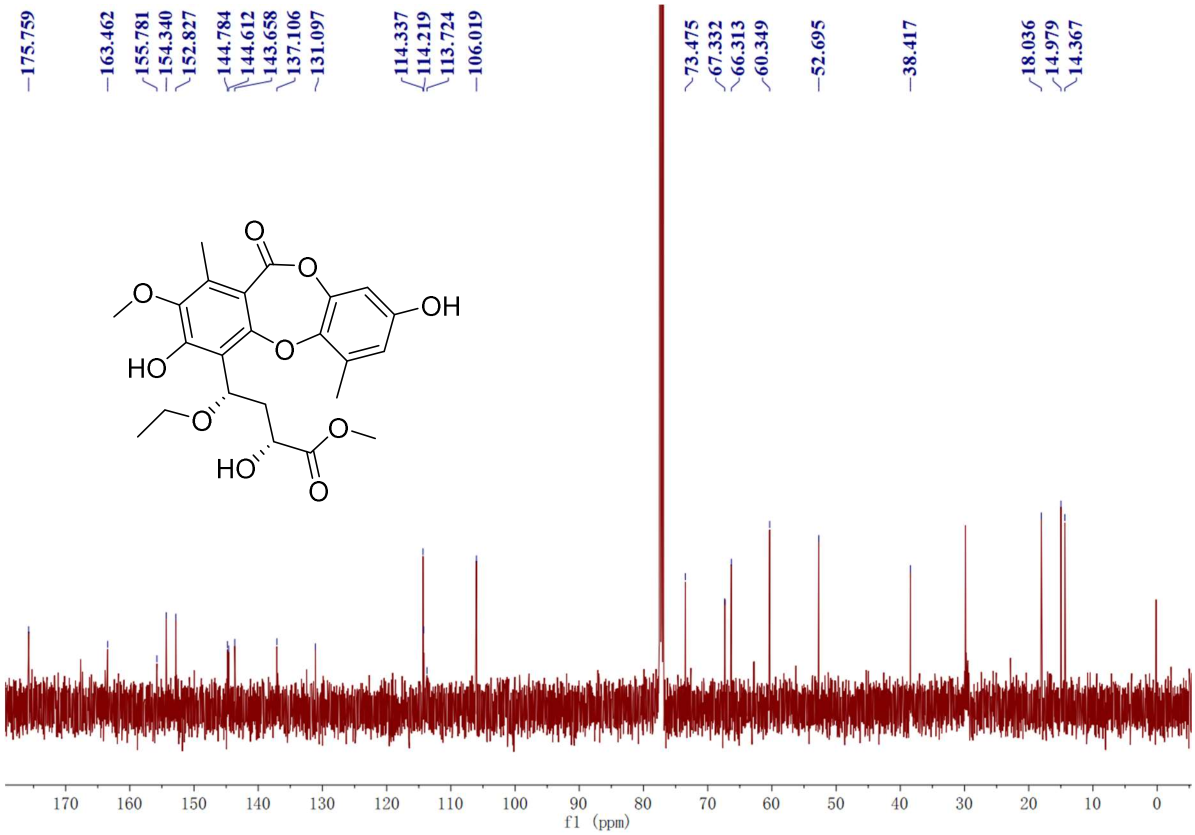

Figure S24. ^{13}C NMR spectra (125 MHz, $CDCl_3$) of **4**.

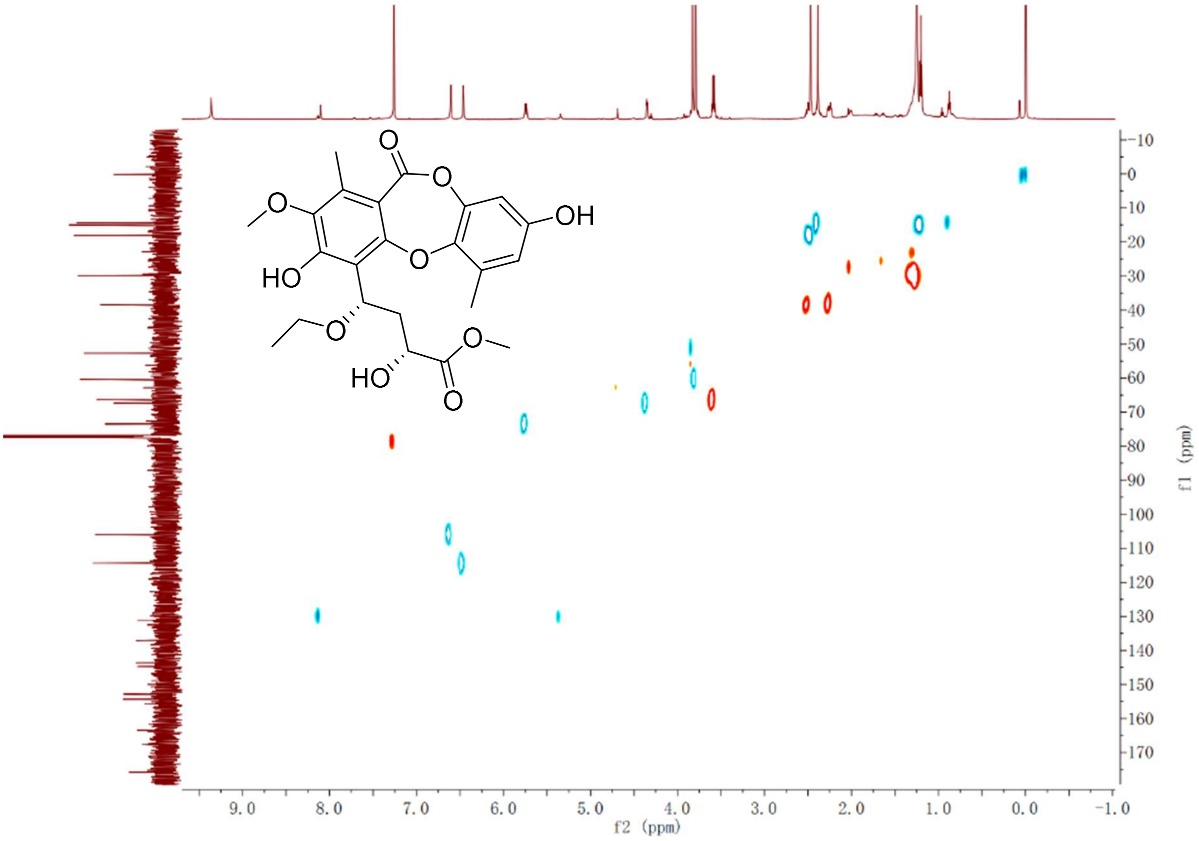

Figure S25. HSQC spectra ($CDCl_3$) of **4**.

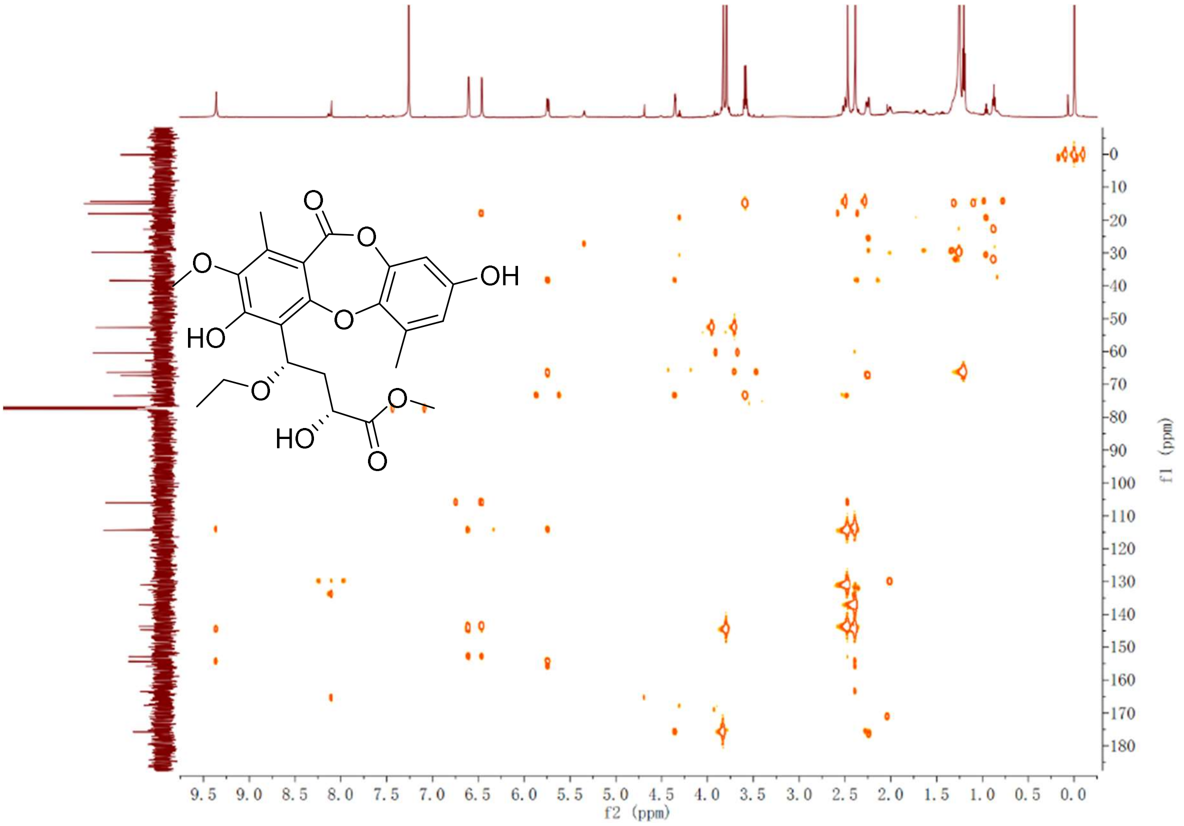

Figure S26. HMBC spectra ($CDCl_3$) of **4**.

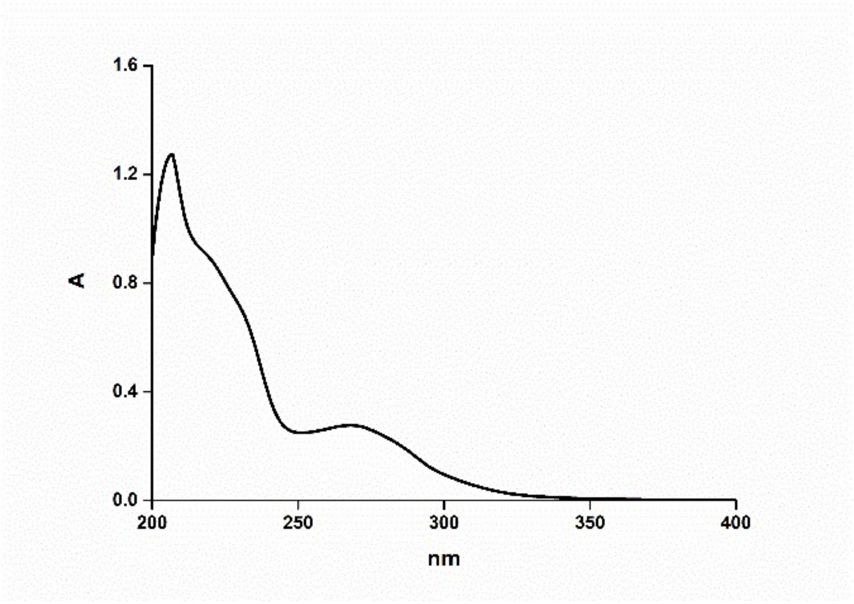

Figure S27. UV spectra (MeOH) of **4**.

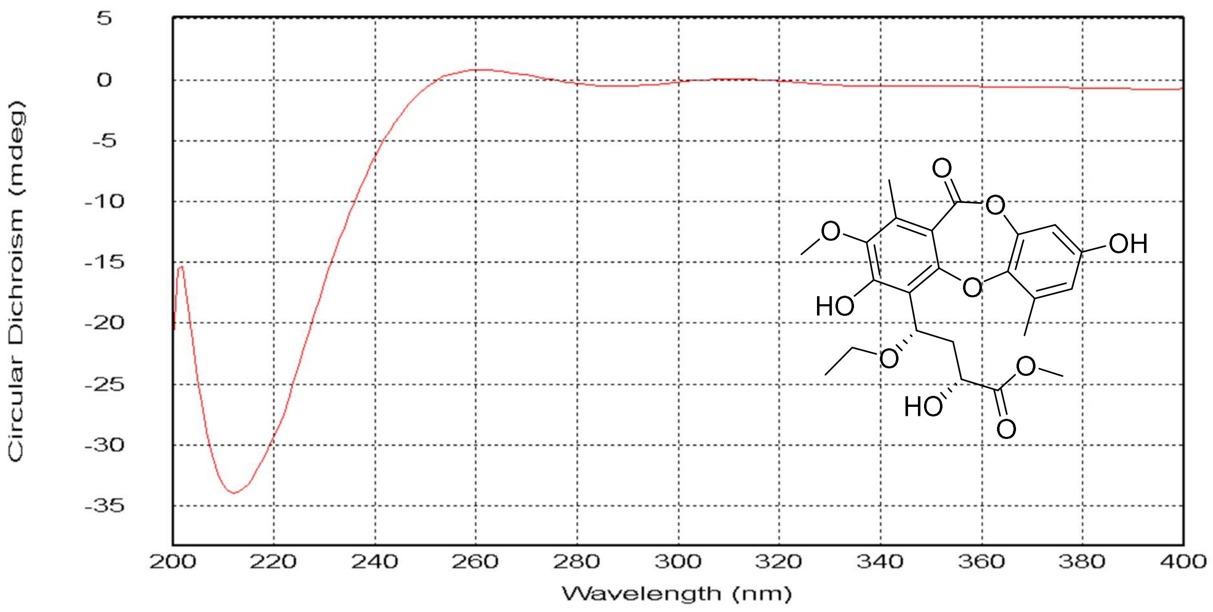

Figure S28. ECD spectra (MeOH) of **4**.

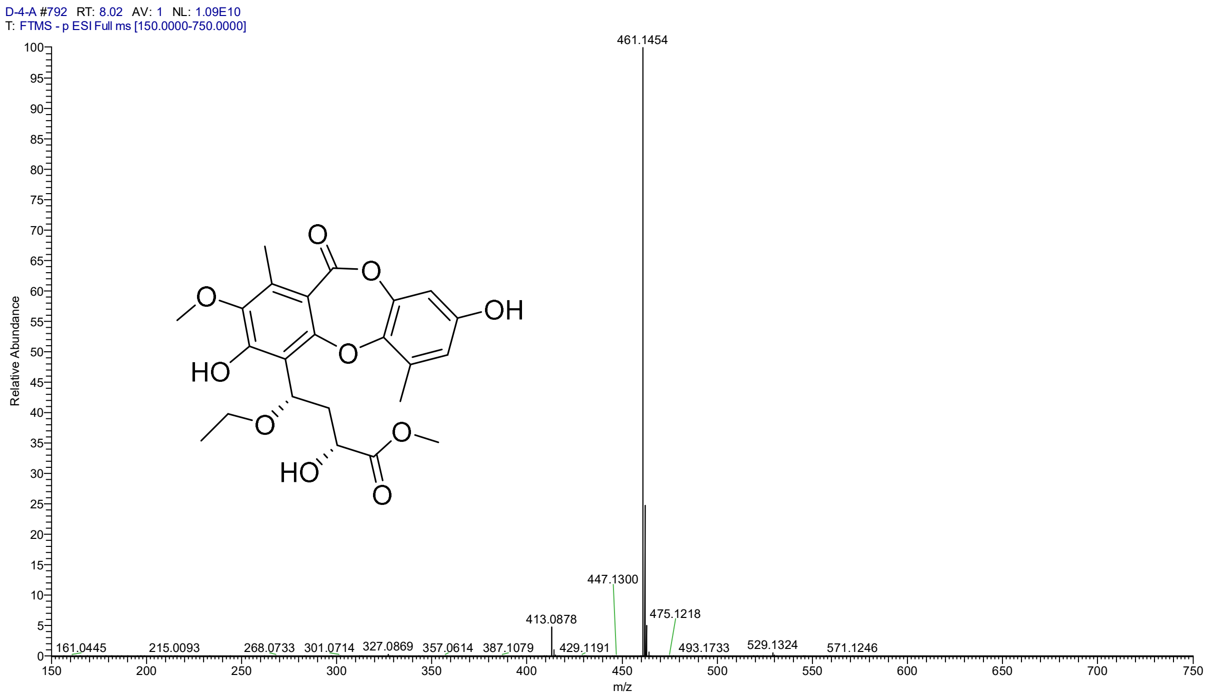

Figure S29. HRESIMS spectra of **4**.

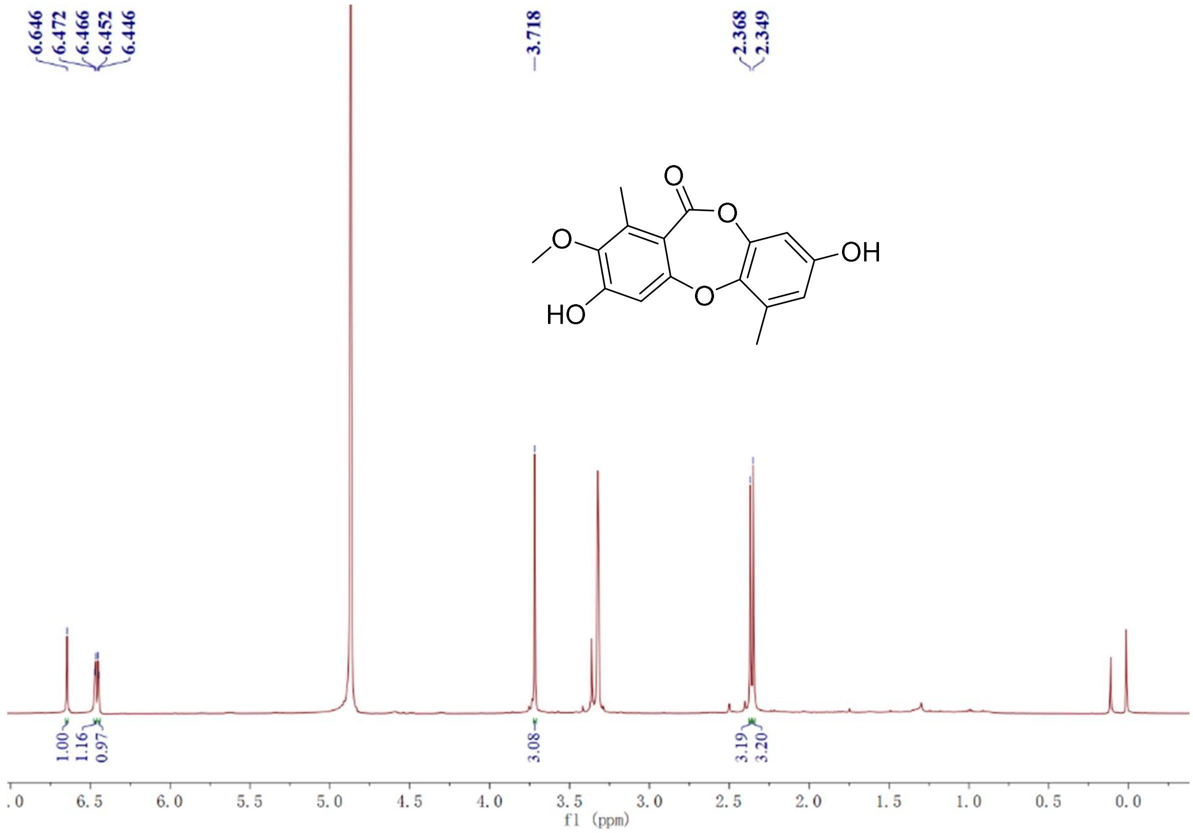

Figure S30. ^{1}H NMR spectra (500 MHz, CD_3OD) of **5**.

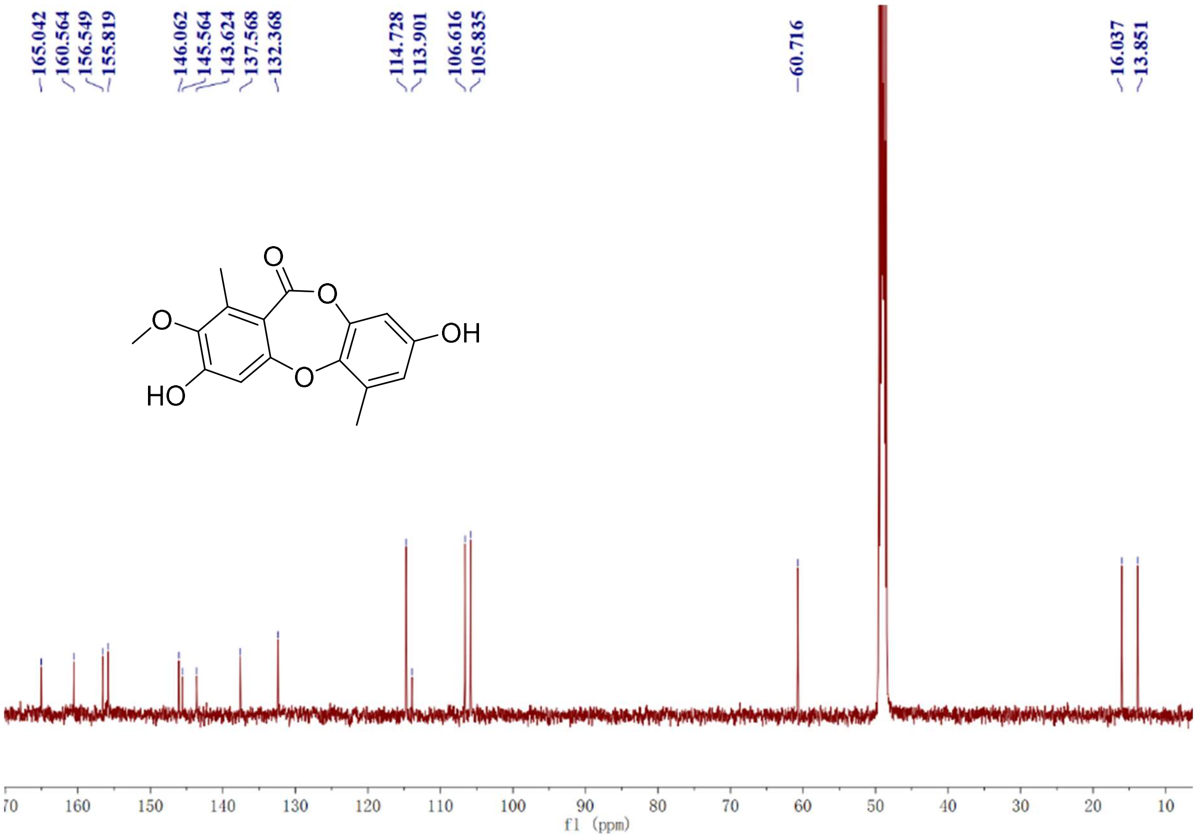

Figure S31. ^{13}C NMR spectra (125 MHz, CD_3OD) of **5**.

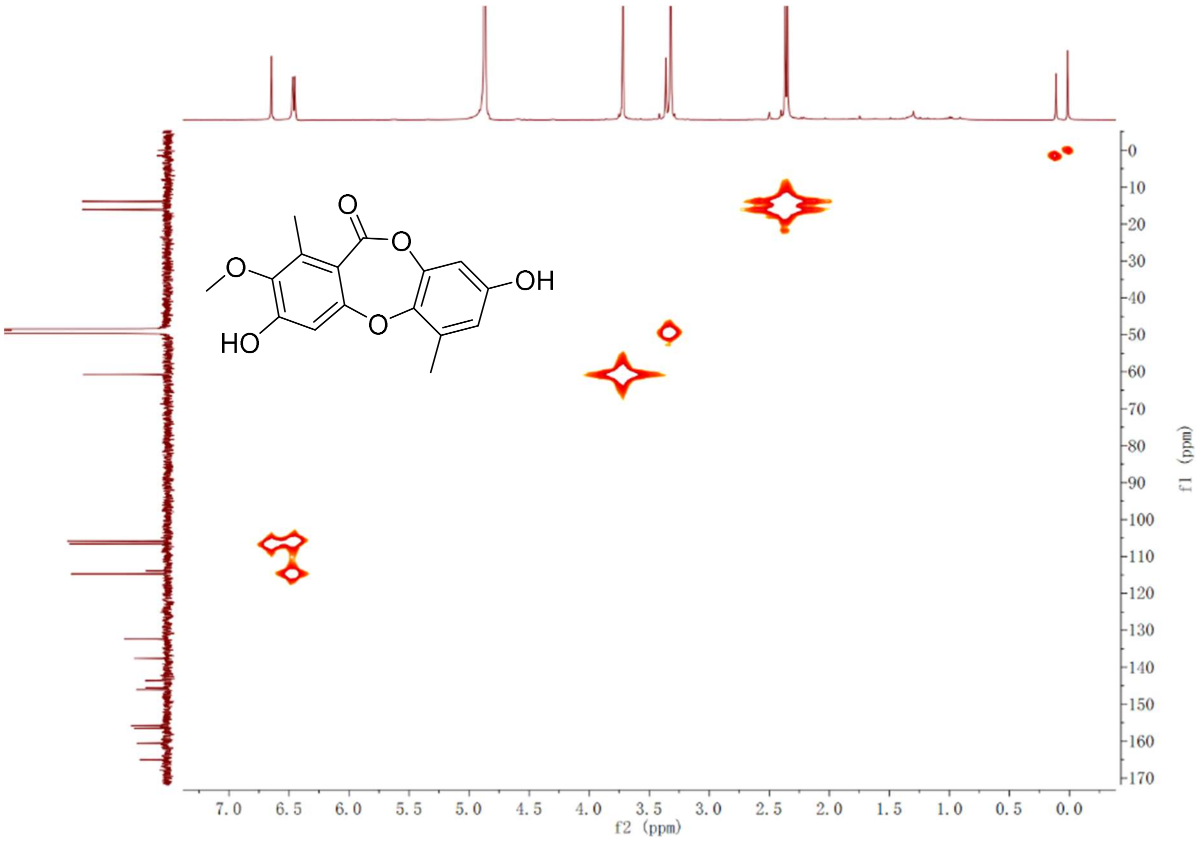

Figure S32. HSQC spectra (CD_3OD) of **5**.

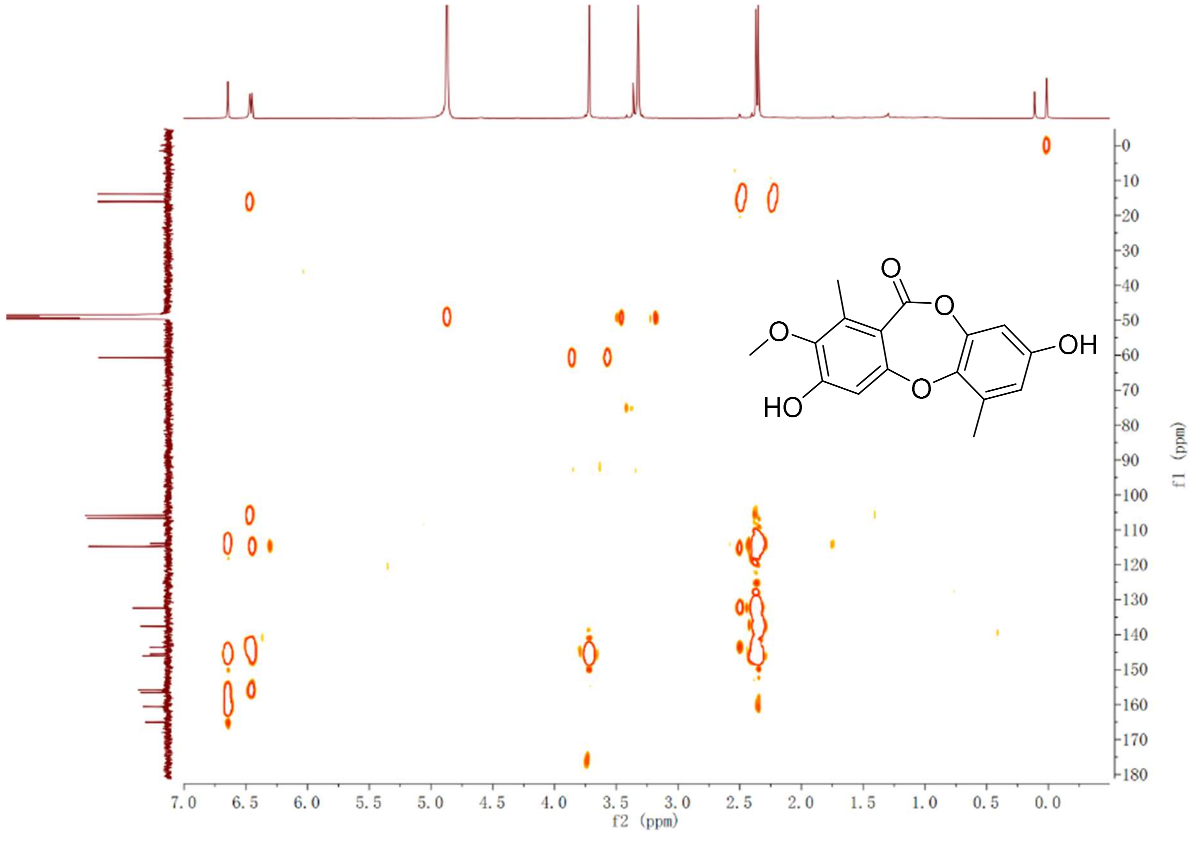

Figure S33. HMBC spectra (CD_3OD) of **5**.

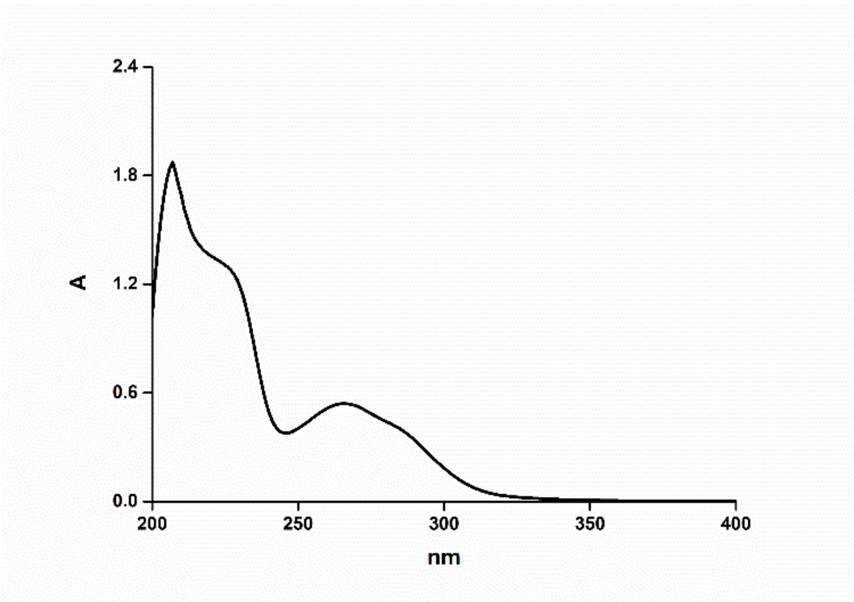

Figure S34. UV spectra (MeOH) of **5**.

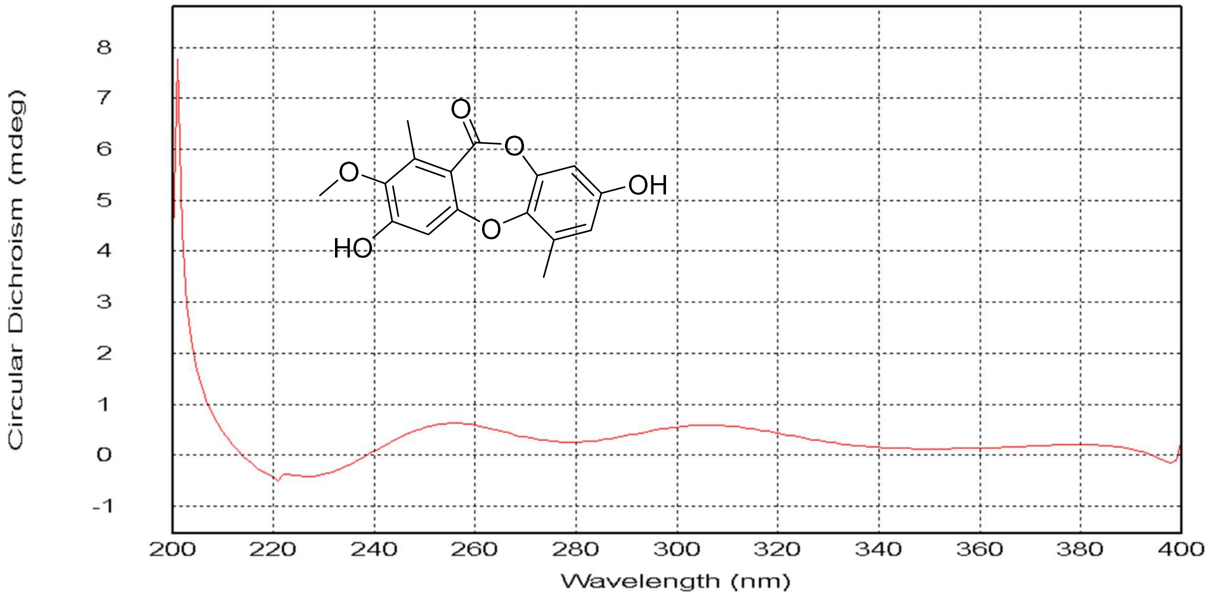

Figure S35. ECD spectra (MeOH) of **5**.

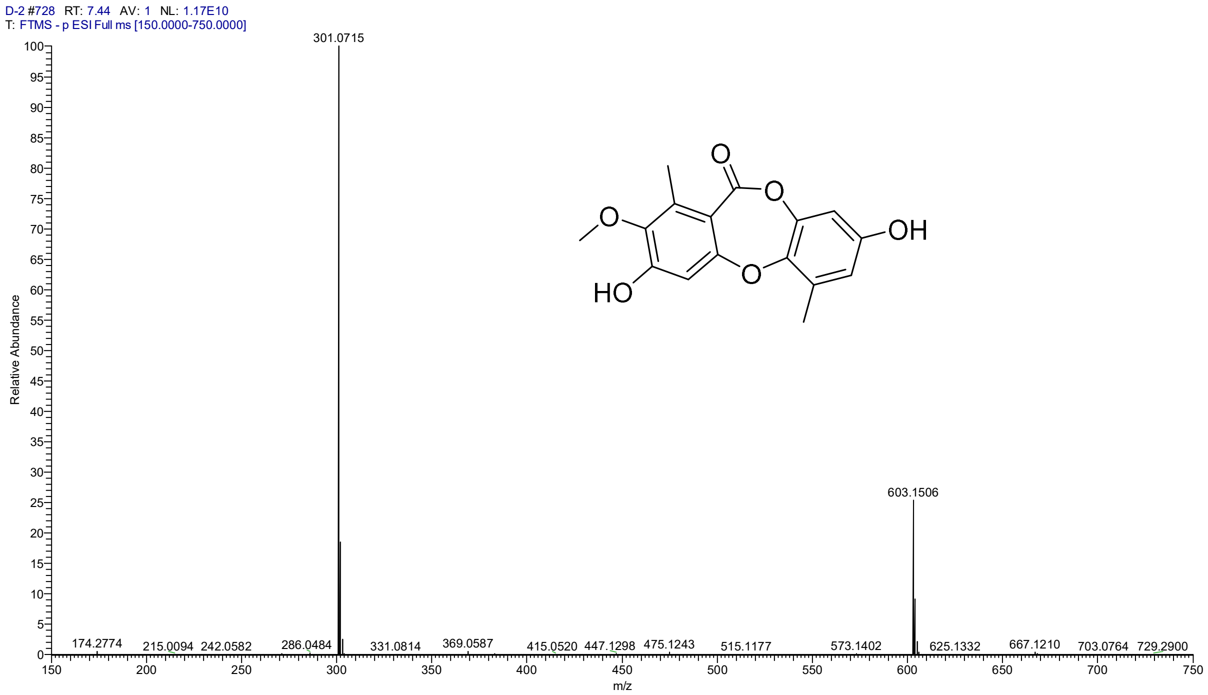

Figure S36. HRESIMS spectra of **5**.

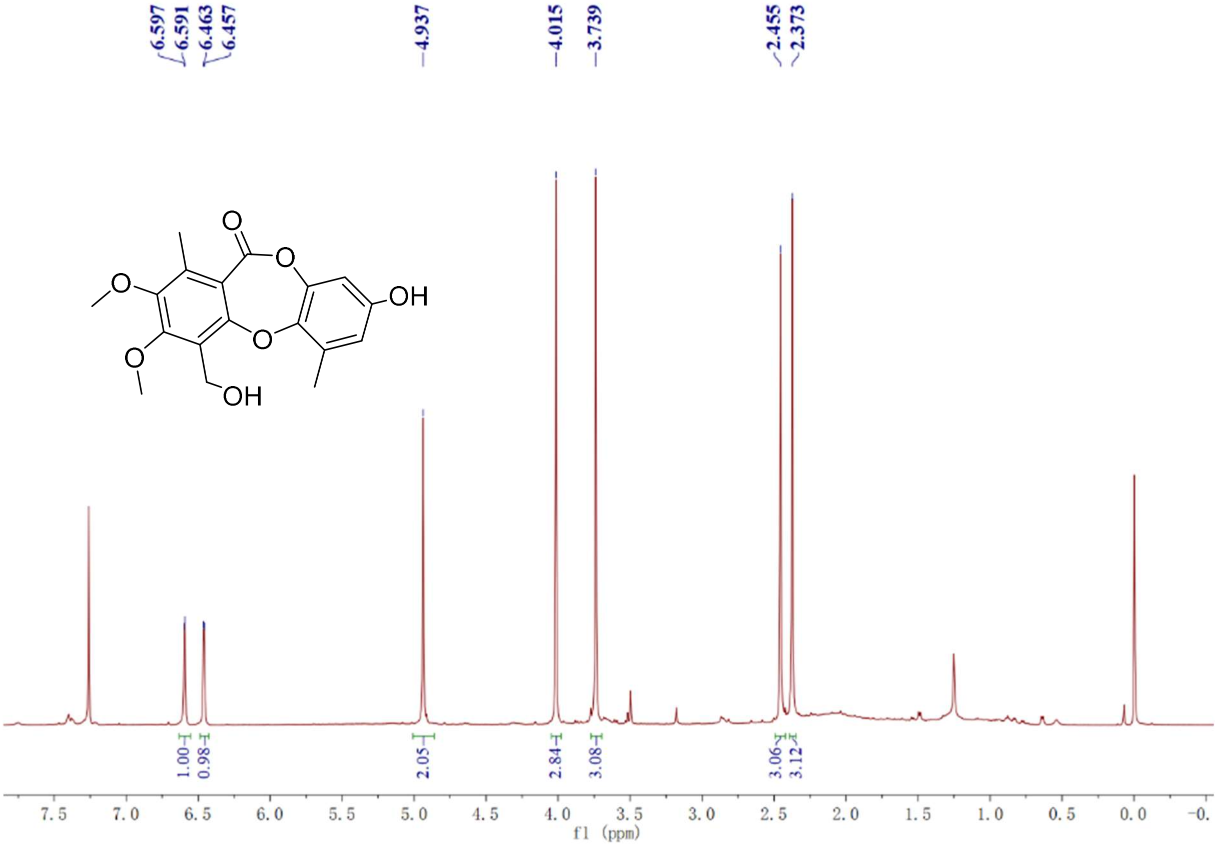

Figure S37. ^{1}H NMR spectra (500 MHz, $CDCl_3$) of **6**.

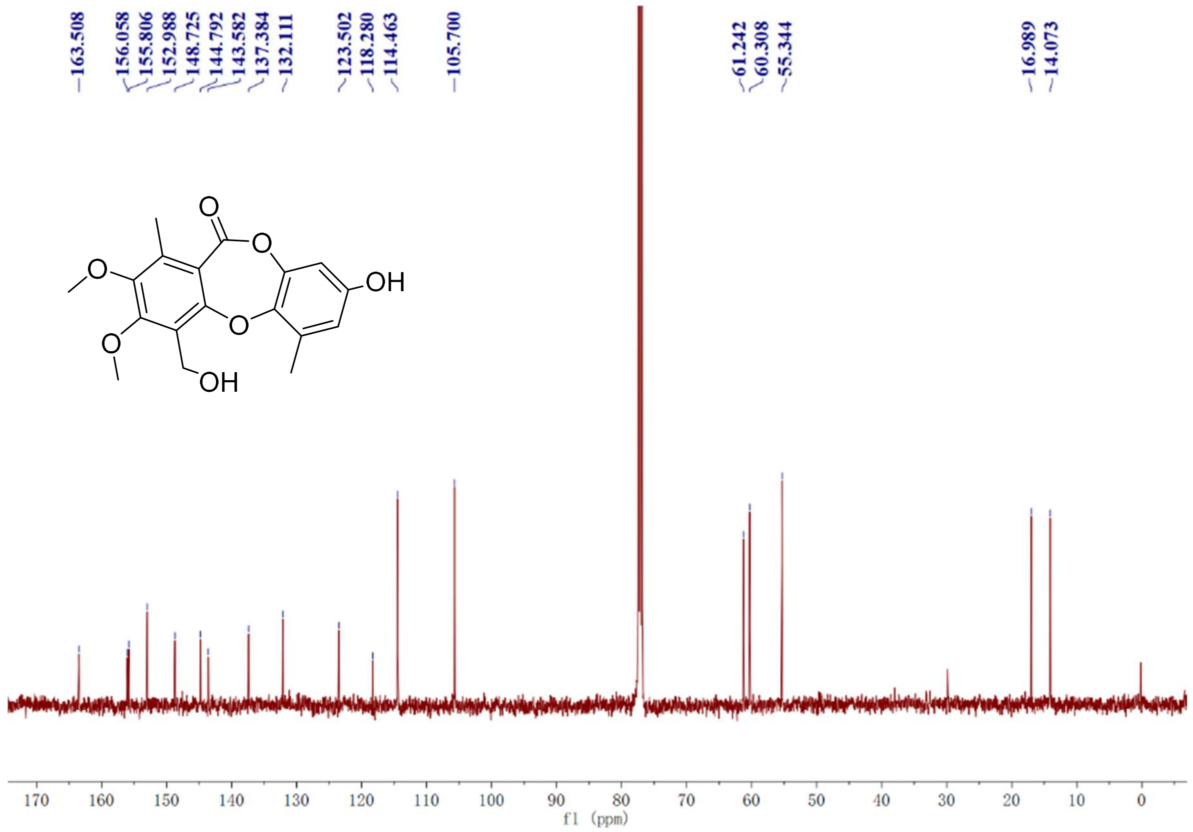

Figure S38. ^{13}C NMR spectra (125 MHz, $CDCl_3$) of **6**.

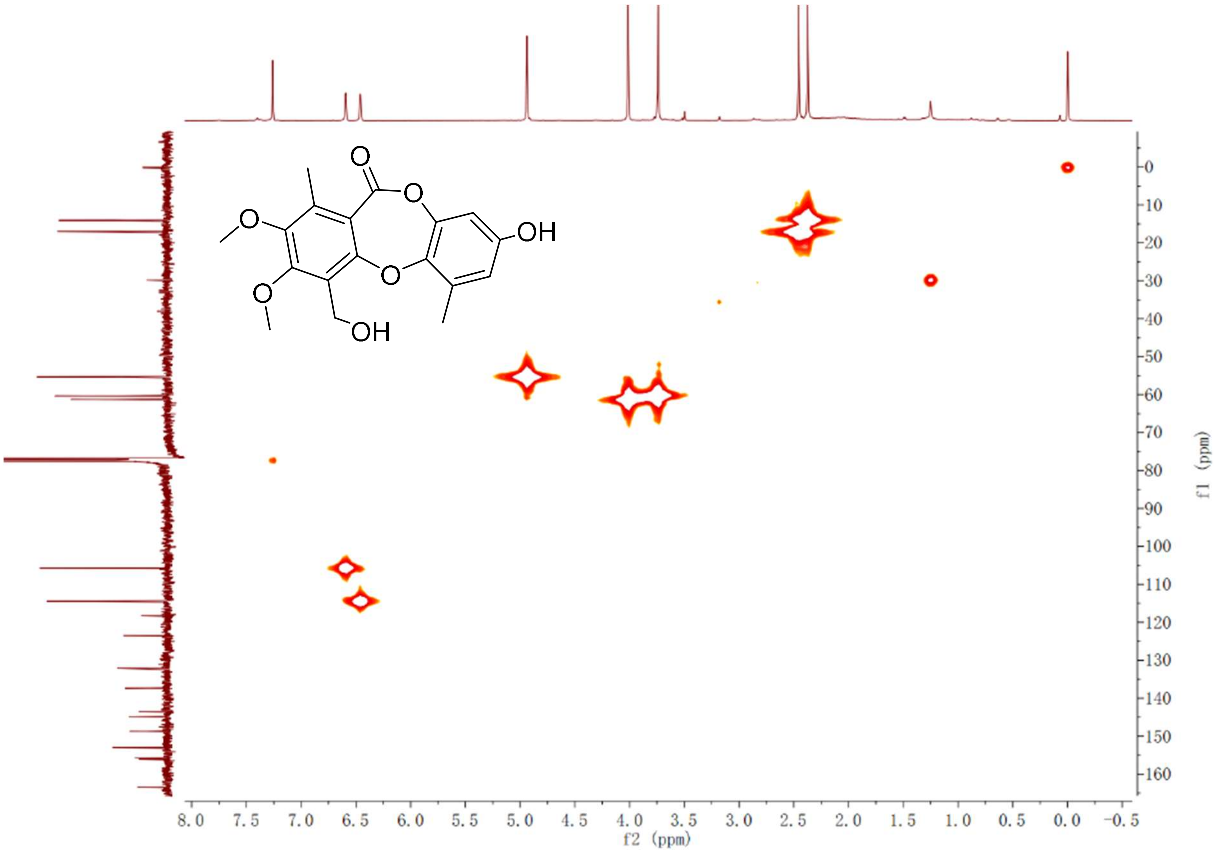

Figure S39. HSQC spectra ($CDCl_3$) of **6**.

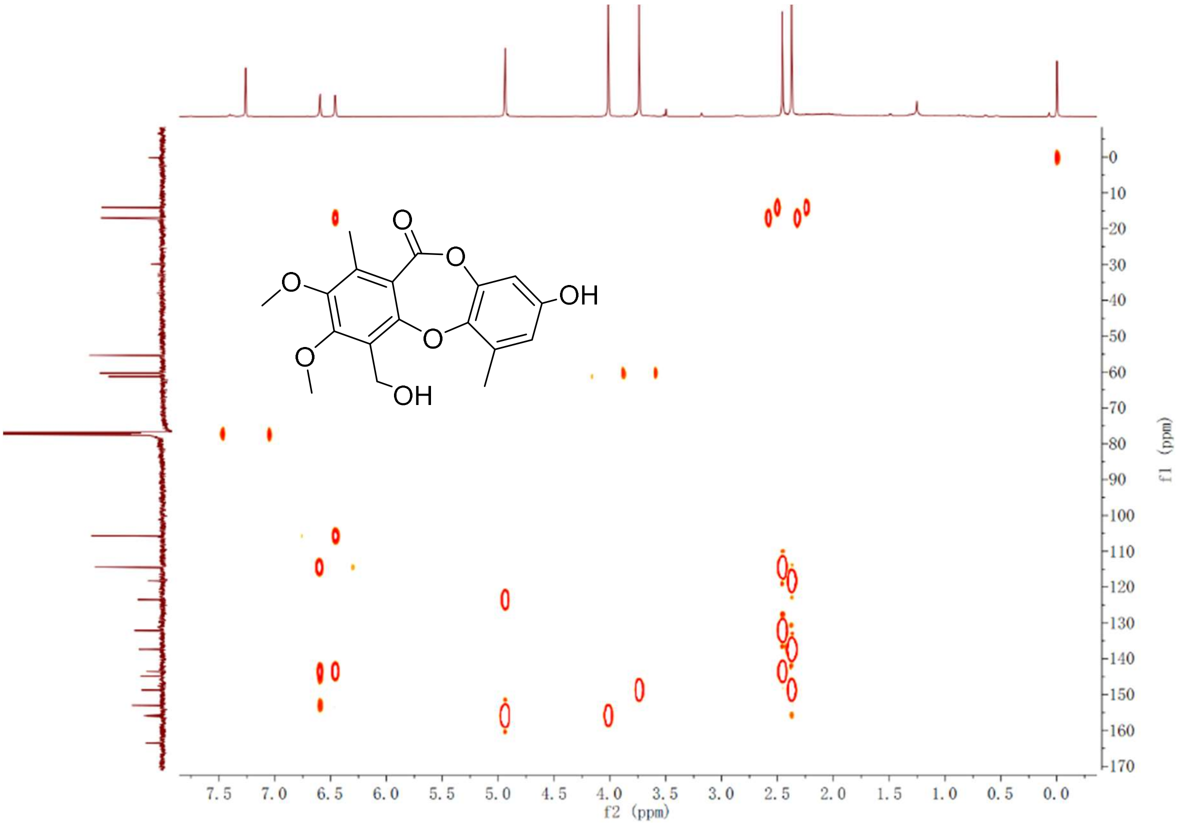

Figure S40. HMBC spectra ($CDCl_3$) of **6**.

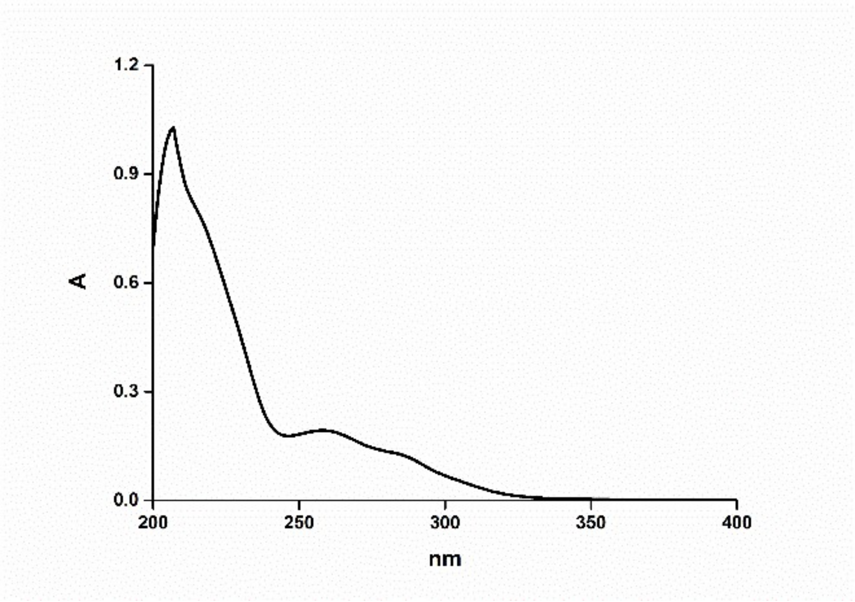

Figure S41. UV spectra (MeOH) of **6**.

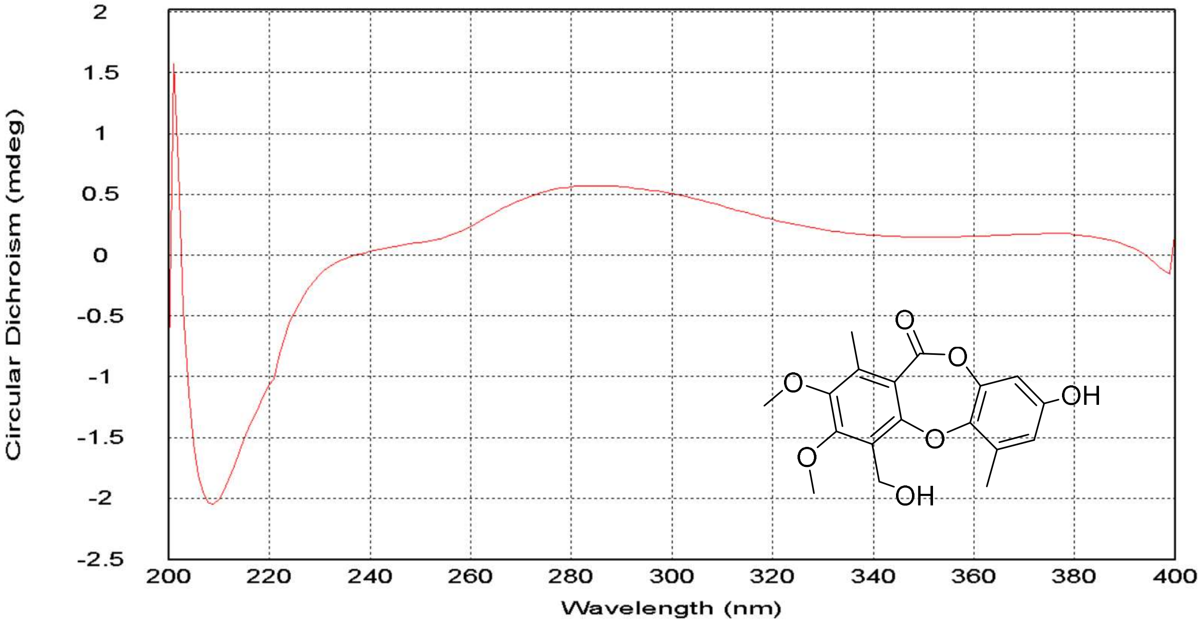

Figure S42. ECD spectra (MeOH) of **6**.

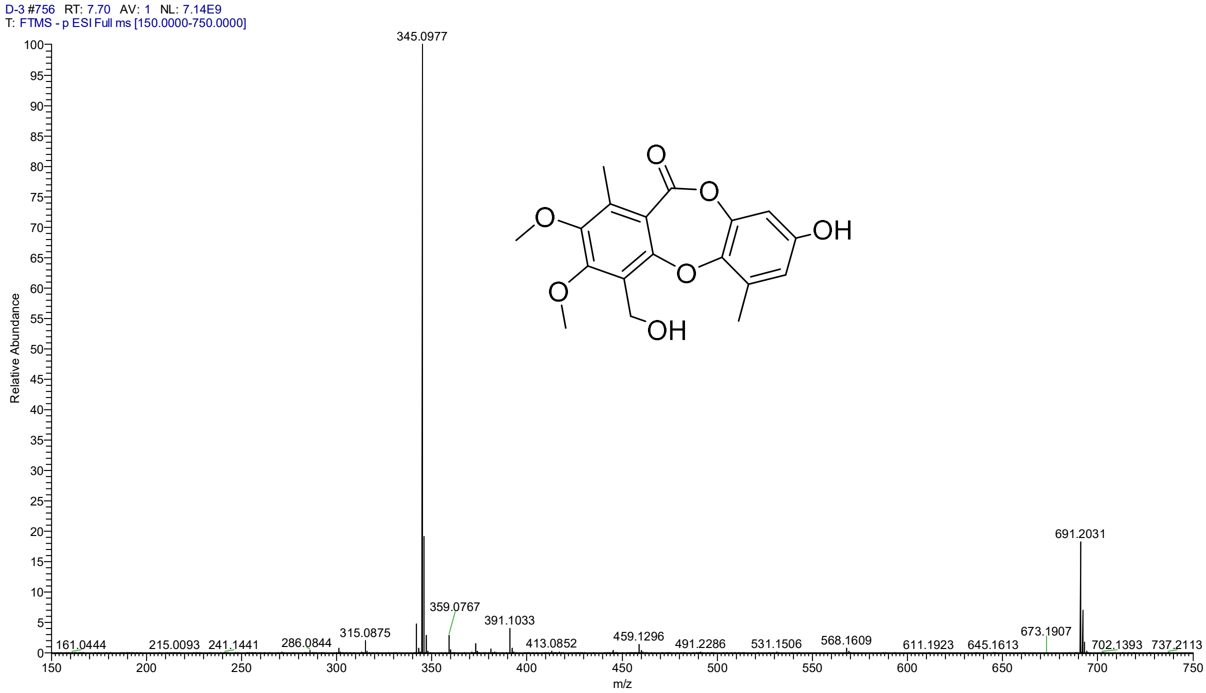

Figure S43. HRESIMS spectra of **6**.

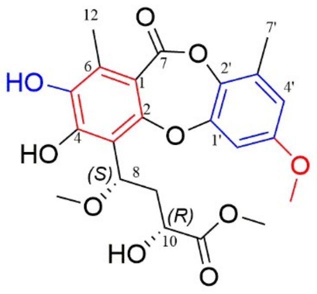

Ⅰa

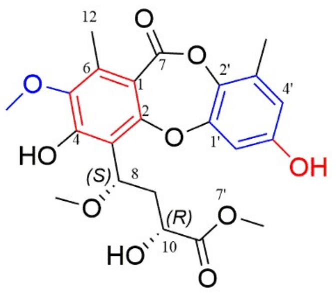

Ⅱa

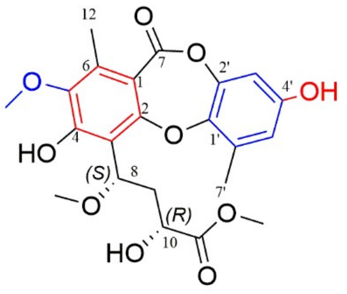

Ⅲa

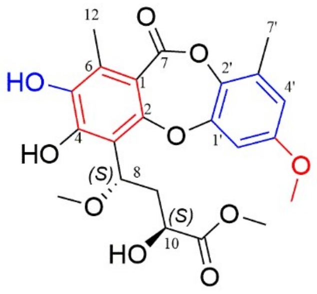

Ⅰb

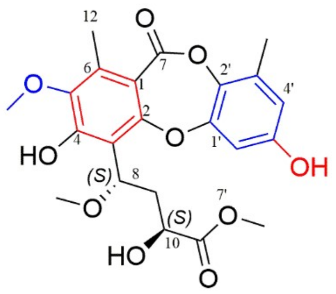

Ⅱb

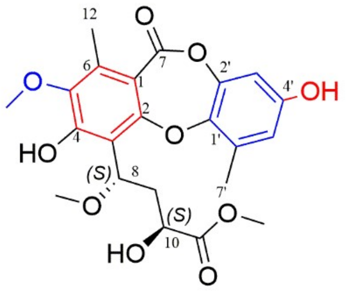

Ⅲb

Figure S44. Chemical structure of undetermined relative configurations of compounds **I**, **II** and **III** (enantiomers not shown).

Table S1. Energies of conformers at MMFF94x force field.

Configuration	Conformer	E (kcal/mol)	ΔE (kcal/mol)
**Ia**	1	100.26	0.00
**Ia**	2	102.31	2.05
**Ia**	3	102.31	2.05
**Ia**	4	102.31	2.05
**Ia**	5	102.31	2.05
**Ia**	6	102.31	2.05
**Ia**	7	102.31	2.05
**Ia**	8	102.31	2.05
**Ia**	9	102.31	2.05
**Ia**	10	102.31	2.05
**Ia**	11	102.31	2.05
**Ia**	12	102.31	2.05
**Ia**	13	102.31	2.05
**Ia**	14	102.72	2.46
**Ia**	15	102.85	2.58
**Ia**	16	102.88	2.62
**Ia**	17	102.92	2.66

**Ia**	18	103.59	3.33
**Ia**	19	103.63	3.36
**Ia**	20	104.22	3.96
**Ia**	21	104.53	4.27
**Ia**	22	104.62	4.36
**Ia**	23	104.64	4.38
**Ia**	24	104.72	4.45
**Ia**	25	104.72	4.45
**Ia**	26	104.72	4.45
**Ia**	27	104.72	4.45
**Ia**	28	104.72	4.45
**Ia**	29	104.72	4.45
**Ia**	30	104.72	4.45
**Ia**	31	104.72	4.45
**Ia**	32	104.72	4.45
**Ia**	33	104.72	4.45
**Ia**	34	104.72	4.45
**Ia**	35	104.72	4.45
**Ia**	36	104.72	4.45
**Ia**	37	104.72	4.45
**Ia**	38	104.72	4.45
**Ia**	39	104.72	4.45
**Ia**	40	104.91	4.64
**Ia**	41	105.05	4.79
**Ia**	42	105.20	4.94
**Ib**	1	101.09	0.00
**Ib**	2	101.22	0.13
**Ib**	3	102.39	1.30
**Ib**	4	102.60	1.51
**Ib**	5	102.98	1.88
**Ib**	6	103.01	1.92
**Ib**	7	103.62	2.52
**Ib**	8	103.62	2.52
**Ib**	9	103.62	2.52
**Ib**	10	103.62	2.52
**Ib**	11	103.62	2.52
**Ib**	12	103.62	2.52
**Ib**	13	103.62	2.52
**Ib**	14	103.62	2.52
**Ib**	15	103.62	2.52
**Ib**	16	103.62	2.52
**Ib**	17	103.62	2.52
**Ib**	18	103.62	2.52

**Ib**	19	103.62	2.52
**Ib**	20	103.62	2.52
**Ib**	21	103.62	2.52
**Ib**	22	103.62	2.52
**Ib**	23	103.62	2.52
**Ib**	24	103.91	2.81
**Ib**	25	104.10	3.01
**Ib**	26	104.46	3.37
**Ib**	27	104.46	3.37
**Ib**	28	104.46	3.37
**Ib**	29	104.46	3.37
**Ib**	30	104.46	3.37
**Ib**	31	104.46	3.37
**Ib**	32	104.46	3.37
**Ib**	33	104.46	3.37
**Ib**	34	104.46	3.37
**Ib**	35	104.46	3.37
**Ib**	36	104.46	3.37
**Ib**	37	104.46	3.37
**Ib**	38	104.46	3.37
**Ib**	39	104.46	3.37
**Ib**	40	104.63	3.53
**Ib**	41	104.73	3.64
**Ib**	42	104.84	3.74
**Ib**	43	104.85	3.76
**Ib**	44	104.94	3.85
**Ib**	45	105.15	4.06
**Ib**	46	105.32	4.22
**Ib**	47	105.41	4.31
**Ib**	48	105.45	4.36
**Ib**	49	105.51	4.41
**Ib**	50	105.64	4.55
**Ib**	51	105.68	4.58
**Ib**	52	105.77	4.67
**Ib**	53	105.83	4.73
**IIa**	1	108.61	0.00
**IIa**	2	108.65	0.04
**IIa**	3	108.72	0.11
**IIa**	4	109.24	0.63
**IIa**	5	110.39	1.77
**IIa**	6	110.94	2.33
**IIa**	7	111.02	2.41
**IIa**	8	111.10	2.48

**IIa**	9	111.16	2.54
**IIa**	10	111.97	3.36
**IIa**	11	112.30	3.69
**IIa**	12	112.46	3.85
**IIa**	13	112.53	3.92
**IIa**	14	112.95	4.34
**IIa**	15	113.07	4.46
**IIa**	16	113.10	4.49
**IIa**	17	113.12	4.51
**IIa**	18	113.20	4.58
**IIa**	19	113.25	4.64
**IIa**	20	113.48	4.86
**IIa**	21	113.60	4.99
**IIb**	1	106.87	0.00
**IIb**	2	109.51	2.64
**IIb**	3	109.89	3.02
**IIb**	4	110.25	3.38
**IIb**	5	110.47	3.60
**IIb**	6	110.48	3.61
**IIb**	7	110.69	3.82
**IIb**	8	111.13	4.26
**IIb**	9	111.16	4.28
**IIb**	10	111.16	4.28
**IIb**	11	111.47	4.60
**IIb**	12	111.50	4.63
**IIb**	13	111.71	4.84
**IIb**	14	111.82	4.95
**IIIa**	1	110.80	0.00
**IIIa**	2	111.52	0.72
**IIIa**	3	112.91	2.12
**IIIa**	4	112.99	2.19
**IIIa**	5	113.23	2.43
**IIIa**	6	113.27	2.48
**IIIa**	7	113.58	2.78
**IIIa**	8	113.72	2.93
**IIIa**	9	113.77	2.97
**IIIa**	10	113.95	3.16
**IIIa**	11	114.26	3.46
**IIIa**	12	114.36	3.56
**IIIa**	13	114.38	3.58
**IIIa**	14	114.52	3.72
**IIIa**	15	114.55	3.75
**IIIa**	16	114.57	3.77

**IIIa**	17	114.62	3.83
**IIIa**	18	115.00	4.20
**IIIa**	19	115.03	4.23
**IIIa**	20	115.04	4.24
**IIIa**	21	115.19	4.39
**IIIa**	22	115.23	4.44
**IIIa**	23	115.45	4.65
**IIIa**	24	115.46	4.66
**IIIa**	25	115.49	4.69
**IIIa**	26	115.49	4.69
**IIIa**	27	115.52	4.72
**IIIa**	28	115.53	4.73
**IIIa**	29	115.56	4.76
**IIIa**	30	115.63	4.83
**IIIa**	31	115.75	4.95
**IIIb**	1	111.67	0.00
**IIIb**	2	111.67	0.00
**IIIb**	3	112.02	0.36
**IIIb**	4	112.19	0.53
**IIIb**	5	112.28	0.62
**IIIb**	6	112.83	1.16
**IIIb**	7	113.24	1.58
**IIIb**	8	113.37	1.71
**IIIb**	9	113.47	1.81
**IIIb**	10	113.47	1.81
**IIIb**	11	113.72	2.05
**IIIb**	12	113.79	2.12
**IIIb**	13	113.79	2.12
**IIIb**	14	113.80	2.13
**IIIb**	15	113.86	2.20
**IIIb**	16	113.88	2.22
**IIIb**	17	113.90	2.23
**IIIb**	18	113.94	2.28
**IIIb**	19	114.15	2.49
**IIIb**	20	114.17	2.50
**IIIb**	21	114.21	2.55
**IIIb**	22	114.22	2.55
**IIIb**	23	114.22	2.55
**IIIb**	24	114.22	2.55
**IIIb**	25	114.26	2.59
**IIIb**	26	114.39	2.73
**IIIb**	27	114.44	2.77
**IIIb**	28	114.45	2.78

**IIIb**	29	114.52	2.86
**IIIb**	30	114.55	2.88
**IIIb**	31	114.68	3.01
**IIIb**	32	114.72	3.06
**IIIb**	33	114.75	3.08
**IIIb**	34	114.75	3.08
**IIIb**	35	114.79	3.12
**IIIb**	36	114.89	3.23
**IIIb**	37	115.04	3.38
**IIIb**	38	115.09	3.42
**IIIb**	39	115.16	3.49
**IIIb**	40	115.24	3.57
**IIIb**	41	115.37	3.71
**IIIb**	42	115.38	3.71
**IIIb**	43	115.47	3.81
**IIIb**	44	115.61	3.95
**IIIb**	45	115.64	3.97
**IIIb**	46	115.65	3.99
**IIIb**	47	115.71	4.04
**IIIb**	48	115.89	4.23
**IIIb**	49	115.92	4.25
**IIIb**	50	115.93	4.27
**IIIb**	51	115.99	4.33
**IIIb**	52	115.99	4.33
**IIIb**	53	116.00	4.34
**IIIb**	54	116.12	4.46
**IIIb**	55	116.31	4.64
**IIIb**	56	116.32	4.65
**IIIb**	57	116.40	4.74
**IIIb**	58	116.51	4.84
**IIIb**	59	116.51	4.85
**IIIb**	60	116.52	4.85
**IIIb**	61	116.52	4.85
**IIIb**	62	116.53	4.87
**IIIb**	63	116.54	4.88
**IIIb**	64	116.56	4.89
**IIIb**	65	116.64	4.98
**IIIb**	66	116.64	4.98

Table S2. Energies of compounds I, II and III at B3LYP/6-311G (d, p) in gas phase.

Configuration	Conformer	Structure	E (Hartree)	E (kcal/mol)	Population (%)
**Ia**	1	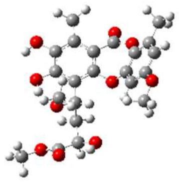	-1605.12910673	-1007233.71	95.47
**Ia**	2	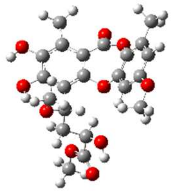	-1605.12622941	-1007231.91	4.53
**Ib**	1	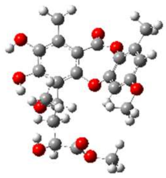	-1605.12656489	-1007232.12	4.85
**Ib**	2	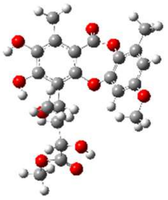	-1605.12937477	-1007233.88	95.15
**IIa**	2	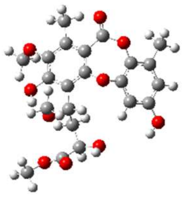	-1605.12216137	-1007229.36	72.45
**IIa**	18	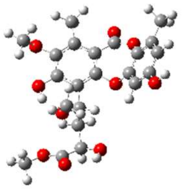	-1605.12124832	-1007228.78	27.55
**IIb**	3	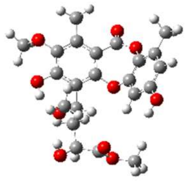	-1605.11814092	-1007226.83	50.11
**IIb**	6	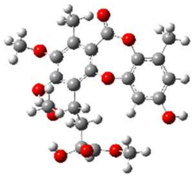	-1605.11813694	-1007226.83	49.89

Table S3. Standard orientations of compounds I, II and III at B3LYP/6-311G (d, p) level in gas phase.

Conformer **Ia**-1

Center Number	Atomic Number	Atomic Type	Coordinates (Angstroms) X	Y	Z
1	6	0	-0.046049	3.349012	-0.727929
2	8	0	0.309909	4.525420	-1.321784
3	1	0	1.227024	4.430017	-1.612412
4	6	0	0.917032	2.334209	-0.617620
5	6	0	0.600659	1.096952	-0.053142
6	6	0	1.631060	-0.012427	0.071685
7	6	0	2.389152	0.023509	1.398091
8	1	0	2.939915	0.961410	1.501739
9	6	0	3.355327	-1.160683	1.561086
10	8	0	2.677572	-2.383403	1.347541
11	1	0	3.264388	-2.914575	0.788078
12	1	0	3.755187	-1.125243	2.585134
13	6	0	4.567791	-1.102546	0.635632
14	8	0	5.259761	0.031391	0.762496
15	6	0	6.438313	0.149442	-0.060309
16	1	0	6.878441	1.108622	0.200474
17	1	0	6.162930	0.125857	-1.115278
18	1	0	7.131446	-0.665486	0.150354
19	1	0	1.650550	-0.022186	2.201418
20	8	0	2.612790	0.066052	-0.991339
21	6	0	2.227451	-0.612881	-2.187999
22	1	0	2.133128	-1.686346	-1.998140
23	1	0	3.021374	-0.447341	-2.915726
24	1	0	1.282631	-0.223492	-2.582036
25	1	0	1.134099	-0.979470	0.001613
26	6	0	-0.697243	0.928439	0.436514
27	6	0	-1.663250	1.937606	0.367074
28	6	0	-1.337470	3.178450	-0.238396
29	6	0	-2.314269	4.317798	-0.387736
30	1	0	-3.325063	3.961437	-0.583885
31	1	0	-1.997994	4.972219	-1.198953
32	1	0	-2.362446	4.908131	0.530684
33	8	0	-0.960779	-0.277579	1.079790
34	6	0	-1.956034	-1.049301	0.496501
35	6	0	-3.254520	-0.570079	0.436252
36	8	0	-3.643282	0.602977	1.058952
37	6	0	-2.975510	1.806086	1.061830
38	6	0	-1.623653	-2.314185	0.015855
39	1	0	-0.602330	-2.651130	0.121399

40	6	0	-2.628598	-3.104938	-0.540595
41	8	0	-2.435814	-4.360917	-1.033388
42	6	0	-1.138408	-4.931446	-0.937674
43	1	0	-0.809645	-5.010129	0.104335
44	1	0	-0.402411	-4.352655	-1.507601
45	1	0	-1.219754	-5.928707	-1.366608
46	6	0	-3.936570	-2.611549	-0.623365
47	1	0	-4.694856	-3.246706	-1.064902
48	6	0	-4.269226	-1.353746	-0.135486
49	6	0	-5.683758	-0.838013	-0.177918
50	1	0	-5.746815	0.096618	-0.741881
51	1	0	-6.050142	-0.620774	0.828707
52	1	0	-6.347891	-1.568139	-0.642184
53	8	0	-3.516676	2.714729	1.630440
54	8	0	2.149208	2.646897	-1.113278
55	1	0	2.659678	1.813404	-1.170698
56	8	0	4.885459	-2.027266	-0.077899

Conformer **Ia**-2

Center Number	Atomic Number	Atomic Type	Coordinates (Angstroms) X	Y	Z
1	6	0	-0.640144	3.307276	0.936507
2	8	0	-1.051106	4.491461	1.475938
3	1	0	-1.724856	4.282159	2.137280
4	6	0	-1.256308	2.123153	1.366575
5	6	0	-0.859166	0.876139	0.874880
6	6	0	-1.530826	-0.397694	1.369316
7	6	0	-2.827369	-0.747679	0.624230
8	1	0	-3.421774	-1.386540	1.283763
9	6	0	-2.539906	-1.532057	-0.668103
10	8	0	-1.882768	-2.748977	-0.366613
11	1	0	-2.490687	-3.453847	-0.636368
12	1	0	-1.903033	-0.936776	-1.330404
13	6	0	-3.831683	-1.836926	-1.415065
14	8	0	-4.455655	-0.734398	-1.835914
15	6	0	-5.690482	-0.937649	-2.556554
16	1	0	-6.036196	0.058155	-2.821549
17	1	0	-6.418770	-1.444760	-1.922801
18	1	0	-5.510681	-1.535695	-3.450324
19	1	0	-3.402414	0.153693	0.401329
20	8	0	-1.884288	-0.271883	2.768220
21	6	0	-0.781311	-0.435959	3.659691
22	1	0	-0.349100	-1.436410	3.550495
23	1	0	-1.174353	-0.321789	4.669661

24	1	0	-0.001875	0.313592	3.483884
25	1	0	-0.845485	-1.240321	1.268445
26	6	0	0.150646	0.864658	-0.090548
27	6	0	0.765822	2.035022	-0.548023
28	6	0	0.370282	3.290126	-0.021231
29	6	0	0.987300	4.602731	-0.434281
30	1	0	2.056987	4.507035	-0.619098
31	1	0	0.820127	5.350177	0.340023
32	1	0	0.539744	4.963552	-1.363651
33	8	0	0.460297	-0.368160	-0.658269
34	6	0	1.759444	-0.807198	-0.447237
35	6	0	2.825370	-0.078271	-0.949803
36	8	0	2.656536	1.007044	-1.792132
37	6	0	1.721569	2.011547	-1.691920
38	6	0	1.952567	-2.004933	0.236921
39	1	0	1.078701	-2.553896	0.557046
40	6	0	3.255068	-2.464737	0.427271
41	8	0	3.575697	-3.624421	1.067513
42	6	0	2.517978	-4.452541	1.530098
43	1	0	1.873027	-4.778208	0.706800
44	1	0	1.909963	-3.942179	2.285987
45	1	0	2.995534	-5.321030	1.980485
46	6	0	4.335222	-1.712844	-0.051802
47	1	0	5.335946	-2.092408	0.115215
48	6	0	4.140636	-0.526318	-0.748441
49	6	0	5.296246	0.257012	-1.313980
50	1	0	5.319264	1.273519	-0.911644
51	1	0	5.208805	0.352326	-2.399372
52	1	0	6.244275	-0.229275	-1.080653
53	8	0	1.751432	2.854808	-2.546312
54	8	0	-2.241311	2.285699	2.296794
55	1	0	-2.408731	1.409670	2.702711
56	8	0	-4.231353	-2.964659	-1.594006

Conformer **Ib**-1

Center Number	Atomic Number	Atomic Type	Coordinates (Angstroms) X	Y	Z
1	6	0	-1.683515	-3.284213	0.508280
2	8	0	-2.066853	-4.503945	0.983914
3	1	0	-1.284918	-4.918513	1.372985
4	6	0	-0.335490	-2.910936	0.615172
5	6	0	0.103614	-1.662891	0.172277
6	6	0	1.561149	-1.255024	0.258466
7	6	0	2.382887	-1.735403	-0.944834

8	1	0	2.404725	-2.829124	-0.962346
9	6	0	3.841879	-1.228086	-0.969112
10	8	0	4.624393	-1.746094	0.082336
11	1	0	4.073500	-1.728923	0.876345
12	1	0	4.304335	-1.555917	-1.901806
13	6	0	3.853758	0.305350	-0.962783
14	8	0	3.951716	0.793740	-2.209374
15	6	0	3.896998	2.228390	-2.342851
16	1	0	4.098686	2.429844	-3.392010
17	1	0	4.647286	2.697446	-1.706120
18	1	0	2.905899	2.594114	-2.069474
19	1	0	1.868910	-1.402725	-1.850709
20	8	0	2.183258	-1.793906	1.460102
21	6	0	1.897786	-1.033123	2.642322
22	1	0	2.321572	-0.029392	2.548352
23	1	0	2.371687	-1.555422	3.472861
24	1	0	0.819798	-0.967681	2.818476
25	1	0	1.618168	-0.169770	0.318796
26	6	0	-0.843161	-0.811880	-0.402340
27	6	0	-2.185585	-1.175424	-0.549729
28	6	0	-2.623097	-2.439795	-0.078784
29	6	0	-4.051184	-2.915901	-0.168434
30	1	0	-4.759917	-2.098003	-0.044476
31	1	0	-4.236527	-3.674383	0.591001
32	1	0	-4.248984	-3.357585	-1.148480
33	8	0	-0.367745	0.400874	-0.894980
34	6	0	-0.916029	1.525063	-0.289198
35	6	0	-2.268817	1.786266	-0.430564
36	8	0	-3.085307	1.032950	-1.256012
37	6	0	-3.130742	-0.340218	-1.345581
38	6	0	-0.077986	2.379890	0.424471
39	1	0	0.973314	2.138480	0.495054
40	6	0	-0.626267	3.526203	1.000799
41	8	0	0.084950	4.442575	1.713887
42	6	0	1.479993	4.229706	1.887838
43	1	0	1.679203	3.301175	2.434050
44	1	0	1.838331	5.075208	2.472268
45	1	0	2.007014	4.201975	0.928282
46	6	0	-1.995975	3.786211	0.865438
47	1	0	-2.392530	4.683395	1.324967
48	6	0	-2.829847	2.933761	0.151988
49	6	0	-4.295630	3.228853	-0.029011
50	1	0	-4.913732	2.430444	0.390794

51	1	0	-4.551620	3.297458	-1.089303
52	1	0	-4.564585	4.166940	0.458009
53	8	0	-3.963244	-0.800971	-2.077374
54	8	0	0.485360	-3.837734	1.189743
55	1	0	1.321880	-3.392178	1.425105
56	8	0	3.749732	0.992805	0.027056

Conformer **Ib**-2

Center Number	Atomic Number	Atomic Type	Coordinates (Angstroms)		
			X	Y	Z
1	6	0	0.449419	3.471274	0.788576
2	8	0	0.340508	4.718286	1.332778
3	1	0	-0.461532	4.723433	1.872650
4	6	0	-0.560026	2.532656	1.054475
5	6	0	-0.483613	1.231393	0.555169
6	6	0	-1.554159	0.192137	0.844965
7	6	0	-2.640905	0.137168	-0.229026
8	1	0	-3.190441	1.081913	-0.259346
9	6	0	-3.614339	-1.029188	0.024809
10	8	0	-2.925065	-2.262159	0.120778
11	1	0	-3.141107	-2.752630	-0.686733
12	1	0	-4.140166	-0.846705	0.966743
13	6	0	-4.642784	-1.118897	-1.093006
14	8	0	-5.459548	-0.062548	-1.126408
15	6	0	-6.442038	-0.052739	-2.185201
16	1	0	-7.004008	0.867484	-2.048050
17	1	0	-7.095280	-0.921758	-2.101514
18	1	0	-5.949037	-0.063635	-3.157699
19	1	0	-2.149213	-0.004512	-1.195377
20	8	0	-2.208503	0.450211	2.109296
21	6	0	-1.517466	-0.090252	3.237214
22	1	0	-2.092703	0.185584	4.120859
23	1	0	-0.503786	0.316842	3.320871
24	1	0	-1.466842	-1.181166	3.163698
25	1	0	-1.091173	-0.793513	0.896887
26	6	0	0.608216	0.920079	-0.259386
27	6	0	1.610694	1.846921	-0.560688
28	6	0	1.539265	3.155483	-0.017042
29	6	0	2.580656	4.219373	-0.259157
30	1	0	3.584736	3.799120	-0.309164
31	1	0	2.539895	4.964330	0.534315
32	1	0	2.403722	4.722901	-1.212816
33	8	0	0.605768	-0.349412	-0.831449
34	6	0	1.674909	-1.158027	-0.471995

35	6	0	2.965155	-0.788231	-0.815412
36	8	0	3.235860	0.297935	-1.629297
37	6	0	2.665612	1.549554	-1.570661
38	6	0	1.412419	-2.349200	0.201337
39	1	0	0.383588	-2.602348	0.414595
40	6	0	2.482102	-3.178753	0.536961
41	8	0	2.356874	-4.371220	1.184526
42	6	0	1.051215	-4.829233	1.507777
43	1	0	0.542355	-4.142120	2.193256
44	1	0	1.186841	-5.791337	1.998715
45	1	0	0.438551	-4.963248	0.609639
46	6	0	3.788727	-2.795300	0.209915
47	1	0	4.599592	-3.458176	0.486478
48	6	0	4.048683	-1.611541	-0.470092
49	6	0	5.447713	-1.217688	-0.865521
50	1	0	5.726746	-0.256717	-0.424676
51	1	0	5.528340	-1.099506	-1.949101
52	1	0	6.167686	-1.969808	-0.540637
53	8	0	3.079946	2.360502	-2.353343
54	8	0	-1.581173	2.984958	1.837525
55	1	0	-2.109901	2.205671	2.106783
56	8	0	-4.685117	-2.046802	-1.867461

Conformer **IIa**-2

Center Number	Atomic Number	Atomic Type	Coordinates (Angstroms)		
			X	Y	Z
1	6	0	0.476520	3.120910	-0.416441
2	8	0	0.280410	4.388540	-0.906041
3	6	0	-0.183180	4.480090	-2.259381
4	1	0	-0.220620	5.544140	-2.491071
5	1	0	-1.179100	4.047211	-2.367641
6	1	0	0.515170	3.984050	-2.942901
7	6	0	-0.632660	2.286820	-0.183451
8	6	0	-0.457620	0.998320	0.341579
9	6	0	-1.632681	0.045441	0.476109
10	6	0	-2.015291	-0.590919	-0.865011
11	1	0	-2.477381	0.158281	-1.512601
12	6	0	-2.951601	-1.803729	-0.727271
13	8	0	-2.337901	-2.823439	0.042779
14	1	0	-2.934131	-2.990489	0.789629
15	1	0	-3.160291	-2.184949	-1.734511
16	6	0	-4.294981	-1.453049	-0.095911
17	8	0	-5.010491	-0.620989	-0.850941
18	6	0	-6.278391	-0.200779	-0.305211

19	1	0	-6.118070	0.366001	0.612369
20	1	0	-6.906201	-1.066468	-0.093471
21	1	0	-6.728580	0.426671	-1.070181
22	1	0	-1.103931	-0.934230	-1.359021
23	8	0	-2.812230	0.728581	0.963289
24	6	0	-2.811900	0.939431	2.377279
25	1	0	-2.843241	-0.019839	2.903449
26	1	0	-3.710980	1.508561	2.611659
27	1	0	-1.929650	1.504331	2.695599
28	1	0	-1.370531	-0.745349	1.180579
29	6	0	0.834920	0.601210	0.682659
30	6	0	1.947650	1.429290	0.505289
31	6	0	1.767360	2.713370	-0.067321
32	6	0	2.914470	3.666510	-0.294171
33	1	0	3.792830	3.150900	-0.684571
34	1	0	3.218320	4.127660	0.647839
35	1	0	2.615960	4.453540	-0.982761
36	8	0	0.974319	-0.655520	1.256999
37	6	0	1.734779	-1.547070	0.511069
38	6	0	3.085999	-1.297900	0.318859
39	8	0	3.735469	-0.244570	0.941519
40	6	0	3.287080	1.052750	1.045329
41	6	0	1.125319	-2.682900	-0.006981
42	1	0	0.064979	-2.843400	0.157069
43	6	0	1.902659	-3.590170	-0.724601
44	8	0	1.377859	-4.730750	-1.262141
45	1	0	0.441259	-4.779660	-1.041871
46	6	0	3.262779	-3.344570	-0.925041
47	1	0	3.842789	-4.066120	-1.487561
48	6	0	3.872289	-2.203620	-0.406411
49	6	0	5.345459	-1.945771	-0.586471
50	1	0	5.849339	-1.866261	0.380249
51	1	0	5.520479	-1.001241	-1.108901
52	1	0	5.811819	-2.748831	-1.158571
53	8	0	4.031900	1.835200	1.569799
54	8	0	-1.862910	2.766961	-0.493231
55	1	0	-2.520140	2.186831	-0.061911
56	8	0	-4.659421	-1.925679	0.956979

Conformer **IIa**-18

Center	Atomic	Atomic	Coordinates (Angstroms)		
Number	Number	Type	X	Y	Z
1	6	0	0.089100	3.028971	0.319230
2	8	0	-0.268940	4.295831	0.711810

3	6	0	-0.528090	4.472811	2.109730
4	1	0	-1.386390	3.880301	2.431040
5	1	0	-0.746570	5.532141	2.242110
6	1	0	0.353080	4.207341	2.705080
7	6	0	-0.877970	2.005081	0.329430
8	6	0	-0.548980	0.709191	-0.098290
9	6	0	-1.594970	-0.393389	-0.152750
10	6	0	-2.402420	-0.364939	-1.451640
11	1	0	-2.925090	0.588321	-1.559560
12	6	0	-3.412560	-1.518749	-1.552260
13	8	0	-2.767660	-2.757369	-1.341060
14	1	0	-3.338380	-3.254459	-0.735790
15	1	0	-3.849650	-1.488499	-2.561480
16	6	0	-4.588620	-1.400489	-0.586220
17	8	0	-5.241750	-0.242229	-0.709290
18	6	0	-6.388410	-0.068319	0.147410
19	1	0	-6.799580	0.903791	-0.112930
20	1	0	-6.082270	-0.089229	1.193970
21	1	0	-7.118370	-0.858639	-0.030570
22	1	0	-1.697150	-0.454159	-2.281180
23	8	0	-2.539310	-0.274599	0.937370
24	6	0	-2.095650	-0.863449	2.159690
25	1	0	-1.158430	-0.412669	2.503910
26	1	0	-1.957590	-1.941669	2.032740
27	1	0	-2.877430	-0.686799	2.897770
28	1	0	-1.111080	-1.366979	-0.083370
29	6	0	0.750120	0.484891	-0.553660
30	6	0	1.711890	1.499491	-0.625160
31	6	0	1.374910	2.801061	-0.173500
32	6	0	2.350250	3.951591	-0.212420
33	1	0	2.445950	4.335121	-1.230220
34	1	0	3.349300	3.643581	0.098690
35	1	0	2.000650	4.758681	0.427300
36	8	0	1.030130	-0.795569	-1.018340
37	6	0	2.039240	-1.453979	-0.331500
38	6	0	3.338690	-0.960729	-0.384920
39	8	0	3.687630	0.101371	-1.198510
40	6	0	3.008840	1.293741	-1.329540
41	6	0	1.736440	-2.610189	0.371980
42	1	0	0.725650	-2.994469	0.390010
43	6	0	2.762550	-3.286419	1.029340
44	8	0	2.426800	-4.424299	1.706800
45	1	0	3.218240	-4.803509	2.102300

46	6	0	4.066460	-2.790058	0.986160
47	1	0	4.862040	-3.320638	1.501250
48	6	0	4.374430	-1.627588	0.276770
49	6	0	5.784860	-1.106488	0.189690
50	1	0	6.115230	-1.046908	-0.850370
51	1	0	6.472870	-1.751388	0.738360
52	1	0	5.858050	-0.095238	0.599030
53	8	0	3.527860	2.125241	-2.022790
54	8	0	-2.119480	2.324191	0.764690
55	1	0	-2.602230	1.484901	0.905430
56	8	0	-4.916720	-2.300229	0.153190

Conformer **IIb**-3

Center Number	Atomic Number	Atomic Type	Coordinates (Angstroms)		
			X	Y	Z
1	6	0	-2.466309	-2.347301	0.326950
2	8	0	-3.260069	-3.379581	0.762320
3	6	0	-3.236358	-4.567151	-0.040620
4	1	0	-3.951068	-5.251811	0.414900
5	1	0	-2.244098	-5.022711	-0.040400
6	1	0	-3.546738	-4.347701	-1.068030
7	6	0	-1.070969	-2.442970	0.494590
8	6	0	-0.239689	-1.370870	0.140410
9	6	0	1.269721	-1.434410	0.290910
10	6	0	1.970011	-2.051200	-0.927270
11	1	0	1.687821	-3.104230	-1.020800
12	6	0	3.512041	-1.970499	-0.883670
13	8	0	4.076691	-2.735879	0.156460
14	1	0	3.499521	-2.635619	0.925680
15	1	0	3.904991	-2.366359	-1.821980
16	6	0	3.947741	-0.502499	-0.787820
17	8	0	4.263681	-0.011399	-1.995930
18	6	0	4.622410	1.384581	-2.048110
19	1	0	3.757600	2.004881	-1.807150
20	1	0	4.943230	1.563181	-3.071380
21	1	0	5.429130	1.594881	-1.345790
22	1	0	1.606081	-1.532680	-1.818370
23	8	0	1.653481	-2.215020	1.457430
24	6	0	1.530621	-1.497590	2.693980
25	1	0	2.218851	-0.647700	2.700270
26	1	0	1.798871	-2.194490	3.487120
27	1	0	0.505271	-1.147980	2.846370
28	1	0	1.646461	-0.424770	0.439040
29	6	0	-0.837719	-0.227630	-0.390270

30	6	0	-2.217789	-0.113881	-0.579350
31	6	0	-3.052549	-1.201181	-0.212460
32	6	0	-4.554219	-1.155281	-0.353410
33	1	0	-4.849629	-1.312301	-1.393020
34	1	0	-4.952919	-0.182291	-0.064090
35	1	0	-5.004729	-1.927381	0.266520
36	8	0	0.025040	0.785890	-0.799090
37	6	0	-0.131950	1.990190	-0.124970
38	6	0	-1.319940	2.696000	-0.246620
39	8	0	-2.320700	2.310699	-1.121430
40	6	0	-2.810730	1.038449	-1.315350
41	6	0	0.924590	2.479100	0.636500
42	1	0	1.847990	1.912860	0.696960
43	6	0	0.776210	3.705660	1.282030
44	8	0	1.767210	4.259220	2.040760
45	1	0	2.539260	3.683890	2.027860
46	6	0	-0.422891	4.413100	1.173450
47	1	0	-0.512041	5.361790	1.688490
48	6	0	-1.479560	3.925849	0.408020
49	6	0	-2.761251	4.700459	0.249390
50	1	0	-2.715711	5.641849	0.798210
51	1	0	-3.616920	4.126729	0.615830
52	1	0	-2.958331	4.917109	-0.803590
53	8	0	-3.726680	0.932869	-2.084560
54	8	0	-0.585369	-3.591380	1.021820
55	1	0	0.347411	-3.436370	1.263740
56	8	0	3.970611	0.147291	0.232950

Conformer **IIb**-6

Center Number	Atomic Number	Atomic Type	Coordinates (Angstroms) X	Y	Z
1	6	0	-2.364201	-2.316779	-0.643600
2	8	0	-3.035287	-3.328192	-1.285890
3	6	0	-3.262493	-4.513873	-0.513140
4	1	0	-2.319171	-4.990709	-0.239380
5	1	0	-3.836250	-5.184165	-1.152390
6	1	0	-3.842314	-4.285855	0.388070
7	6	0	-0.976141	-2.437314	-0.443320
8	6	0	-0.242765	-1.379961	0.116680
9	6	0	1.270736	-1.464056	0.235700
10	6	0	1.970644	-1.161573	-1.097160
11	1	0	1.611337	-1.848234	-1.868390
12	6	0	3.511985	-1.282337	-1.059880
13	8	0	3.953090	-2.616575	-1.000300

14	1	0	3.534971	-3.004737	-0.220430
15	1	0	3.906693	-0.863356	-1.987790
16	6	0	4.088642	-0.464805	0.101020
17	8	0	4.164287	0.843515	-0.216980
18	6	0	4.686843	1.721227	0.803410
19	1	0	4.117974	1.615505	1.727660
20	1	0	4.584720	2.725177	0.398500
21	1	0	5.734004	1.485681	0.997400
22	1	0	1.686011	-0.151714	-1.402020
23	8	0	1.686511	-2.791204	0.652480
24	6	0	1.553391	-3.022565	2.060310
25	1	0	2.225569	-2.358742	2.611020
26	1	0	1.840265	-4.058933	2.235280
27	1	0	0.520971	-2.868308	2.389710
28	1	0	1.603013	-0.754524	0.995850
29	6	0	-0.936959	-0.229064	0.491670
30	6	0	-2.324960	-0.106959	0.351700
31	6	0	-3.051846	-1.171262	-0.242270
32	6	0	-4.541386	-1.106188	-0.477450
33	1	0	-4.852200	-0.111569	-0.798350
34	1	0	-5.088975	-1.319230	0.443190
35	1	0	-4.824053	-1.833509	-1.235750
36	8	0	-0.180003	0.789829	1.058470
37	6	0	-0.230388	2.011109	0.401770
38	6	0	-1.429660	2.712314	0.345690
39	8	0	-2.562099	2.281450	1.011720
40	6	0	-3.081424	1.005758	0.993310
41	6	0	0.931580	2.527873	-0.151810
42	1	0	1.858763	1.972777	-0.113870
43	6	0	0.888236	3.782323	-0.757620
44	8	0	2.058694	4.265617	-1.272430
45	1	0	1.897931	5.123657	-1.678270
46	6	0	-0.314387	4.487378	-0.822310
47	1	0	-0.343071	5.463508	-1.297710
48	6	0	-1.486995	3.965744	-0.272230
49	6	0	-2.782928	4.732539	-0.304740
50	1	0	-3.553526	4.181076	-0.850340
51	1	0	-2.649172	5.703120	-0.784490
52	1	0	-3.168088	4.891018	0.705530
53	8	0	-4.149233	0.859644	1.522470
54	8	0	-0.388136	-3.592152	-0.832460
55	1	0	0.504274	-3.617908	-0.436970
56	8	0	4.401823	-0.917834	1.172870

Conformer **IIIa**-9					
Center	Atomic	Atomic	Coordinates (Angstroms)		
Number	Number	Type	X	Y	Z
1	6	0	0.435483	3.258198	0.495900
2	8	0	0.385676	4.547098	0.967600
3	6	0	-0.476028	5.441893	0.251100
4	1	0	-0.176929	5.512095	-0.800400
5	1	0	-0.359533	6.415594	0.726000
6	1	0	-1.518127	5.124088	0.321200
7	6	0	-0.646913	2.394693	0.750400
8	6	0	-0.582306	1.037493	0.392200
9	6	0	-1.805102	0.141587	0.549000
10	6	0	-2.771803	0.343882	-0.625300
11	1	0	-3.081708	1.391281	-0.658100
12	6	0	-4.084199	-0.473024	-0.574200
13	8	0	-5.047902	0.116271	-1.423200
14	1	0	-5.087699	-0.436929	-2.216500
15	1	0	-4.464499	-0.459826	0.450000
16	6	0	-3.880692	-1.920723	-1.002500
17	8	0	-3.248388	-2.665020	-0.082200
18	6	0	-3.042181	-4.051119	-0.438300
19	1	0	-2.379981	-4.126216	-1.301400
20	1	0	-3.995979	-4.522924	-0.674100
21	1	0	-2.590079	-4.511517	0.436500
22	1	0	-2.240402	0.140985	-1.559500
23	8	0	-2.548604	0.463083	1.748800
24	6	0	-1.999401	-0.075514	2.949500
25	1	0	-0.964503	0.251491	3.101400
26	1	0	-2.039695	-1.169314	2.929400
27	1	0	-2.616403	0.291383	3.769400
28	1	0	-1.496197	-0.901212	0.601300
29	6	0	0.587696	0.578199	-0.221700
30	6	0	1.627792	1.448904	-0.589700
31	6	0	1.556485	2.812803	-0.198400
32	6	0	2.665580	3.799209	-0.475200
33	1	0	2.606576	4.619509	0.237800
34	1	0	2.582178	4.203109	-1.486400
35	1	0	3.647082	3.330414	-0.407300
36	8	0	0.633703	-0.775801	-0.528600
37	6	0	1.776106	-1.467395	-0.123600
38	6	0	3.007704	-1.150589	-0.681400
39	8	0	3.138300	-0.210289	-1.676800
40	6	0	2.640493	1.075409	-1.615000

41	6	0	1.674311	-2.526596	0.787700
42	6	0	0.348713	-2.912603	1.389600
43	1	0	0.418718	-3.888302	1.872700
44	1	0	-0.435087	-2.954906	0.632300
45	1	0	0.033910	-2.188204	2.145900
46	6	0	2.826515	-3.235390	1.123100
47	1	0	2.775819	-4.063991	1.818800
48	6	0	4.065113	-2.900984	0.574500
49	8	0	5.143517	-3.641279	0.961700
50	1	0	5.930515	-3.324075	0.506900
51	6	0	4.158208	-1.853084	-0.335900
52	1	0	5.094907	-1.575879	-0.805800
53	8	0	3.046290	1.836511	-2.449400
54	8	0	-1.727516	2.929987	1.359900
55	1	0	-2.317012	2.193984	1.622000
56	8	0	-4.251990	-2.343325	-2.071900

Conformer **IIIa**-14

Center	Atomic	Atomic	Coordinates (Angstroms)		
Number	Number	Type	X	Y	Z
1	6	0	0.685863	3.287587	0.346000
2	8	0	0.757290	4.604685	0.730200
3	6	0	-0.038491	5.521102	-0.032600
4	1	0	0.174929	6.511297	0.369100
5	1	0	-1.102796	5.304024	0.078400
6	1	0	0.240008	5.491296	-1.091800
7	6	0	-0.461252	2.540310	0.676400
8	6	0	-0.516881	1.162411	0.412300
9	6	0	-1.793097	0.369338	0.658200
10	6	0	-2.753495	0.487157	-0.532200
11	1	0	-3.074873	1.526364	-0.641300
12	6	0	-4.041212	-0.359916	-0.410600
13	8	0	-5.009604	0.041604	-1.360600
14	1	0	-4.776812	-0.362101	-2.204200
15	1	0	-4.476109	-0.192407	0.575100
16	6	0	-3.692043	-1.841523	-0.500700
17	8	0	-3.908952	-2.325219	-1.738000
18	6	0	-3.556281	-3.707426	-1.956700
19	1	0	-3.876786	-3.932019	-2.970900
20	1	0	-4.069494	-4.344915	-1.236900
21	1	0	-2.478484	-3.840448	-1.855900
22	1	0	-2.216000	0.215646	-1.446000
23	8	0	-2.515587	0.856253	1.813300
24	6	0	-2.044598	0.342043	3.059700

25	1	0	-2.637988	0.818455	3.839600
26	1	0	-0.985793	0.577821	3.216100
27	1	0	-2.192620	-0.741154	3.100900
28	1	0	-1.545219	-0.677167	0.824000
29	6	0	0.593107	0.563789	-0.192700
30	6	0	1.694922	1.314366	-0.637600
31	6	0	1.749251	2.702065	-0.334500
32	6	0	2.932969	3.568341	-0.691800
33	1	0	2.850376	3.935942	-1.717100
34	1	0	3.869157	3.013921	-0.634500
35	1	0	2.974686	4.421340	-0.016500
36	8	0	0.505679	-0.803810	-0.413500
37	6	0	1.591563	-1.577532	0.001200
38	6	0	2.824966	-1.408857	-0.612900
39	8	0	3.002784	-0.541061	-1.667100
40	6	0	2.640712	0.790047	-1.660200
41	6	0	1.424643	-2.565828	0.981600
42	6	0	0.097338	-2.789701	1.657600
43	1	0	-0.144845	-1.959296	2.326900
44	1	0	0.126819	-3.699302	2.259100
45	1	0	-0.723064	-2.880684	0.944400
46	6	0	2.521626	-3.356751	1.320700
47	1	0	2.423010	-4.132649	2.069900
48	6	0	3.763830	-3.173377	0.712400
49	8	0	4.784413	-3.987498	1.110400
50	1	0	5.580918	-3.767214	0.616600
51	6	0	3.919550	-2.193480	-0.262800
52	1	0	4.859854	-2.031599	-0.777400
53	8	0	3.097325	1.463737	-2.543100
54	8	0	-1.480839	3.203631	1.264300
55	1	0	-2.125253	2.535945	1.578100
56	8	0	-3.223956	-2.491833	0.402800

Conformer **IIIa**-26

Center Number	Atomic Number	Atomic Type	Coordinates (Angstroms)		
			X	Y	Z
1	6	0	-0.832814	2.979488	0.501404
2	8	0	-1.357816	4.196287	0.864704
3	6	0	-2.666616	4.486485	0.358504
4	1	0	-3.405215	3.786984	0.755404
5	1	0	-2.676316	4.457985	-0.736796
6	1	0	-2.902918	5.495385	0.695604
7	6	0	-1.319113	1.812787	1.119304
8	6	0	-0.729211	0.562188	0.862304

9	6	0	-1.300709	-0.695813	1.505604
10	6	0	-2.513008	-1.261814	0.748404
11	1	0	-3.060508	-1.909015	1.439604
12	6	0	-2.088107	-2.109714	-0.462696
13	8	0	-1.356106	-3.244713	-0.041296
14	1	0	-1.907705	-4.013414	-0.251096
15	1	0	-1.463208	-1.512813	-1.135096
16	6	0	-3.307207	-2.571916	-1.251096
17	8	0	-3.973708	-1.558516	-1.809496
18	6	0	-5.145608	-1.914018	-2.574996
19	1	0	-4.872907	-2.582218	-3.392196
20	1	0	-5.535009	-0.974419	-2.958196
21	1	0	-5.879707	-2.405619	-1.935796
22	1	0	-3.176710	-0.454515	0.430504
23	8	0	-1.765610	-0.427913	2.851104
24	6	0	-0.725410	-0.320412	3.822604
25	1	0	-1.204910	-0.066013	4.767404
26	1	0	-0.003911	0.460789	3.559404
27	1	0	-0.203408	-1.276911	3.930104
28	1	0	-0.536908	-1.469712	1.554804
29	6	0	0.338489	0.517589	-0.040396
30	6	0	0.780788	1.658590	-0.728696
31	6	0	0.195986	2.917889	-0.434696
32	6	0	0.662784	4.204190	-1.072396
33	1	0	1.741084	4.205891	-1.231196
34	1	0	0.389883	5.044490	-0.436696
35	1	0	0.203684	4.340089	-2.054096
36	8	0	0.873191	-0.735410	-0.309096
37	6	0	2.252591	-0.884408	-0.162296
38	6	0	3.119590	-0.145507	-0.958696
39	8	0	2.681389	0.656993	-1.988796
40	6	0	1.646288	1.561991	-1.935696
41	6	0	2.761692	-1.843607	0.724104
42	6	0	1.846694	-2.728909	1.531004
43	1	0	1.474493	-2.203709	2.416404
44	1	0	2.386695	-3.610808	1.879104
45	1	0	0.983894	-3.054610	0.947804
46	6	0	4.144393	-1.993205	0.823304
47	1	0	4.566194	-2.731405	1.494504
48	6	0	5.012691	-1.213004	0.060204
49	8	0	6.352492	-1.413602	0.228504
50	1	0	6.839191	-0.836702	-0.368996
51	6	0	4.499090	-0.289505	-0.844796

52	1	0	5.136289	0.301496	-1.492796
53	8	0	1.492987	2.243891	-2.912296
54	8	0	-2.356813	1.957886	1.973304
55	1	0	-2.440512	1.122286	2.478704
56	8	0	-3.621405	-3.736116	-1.343896

Conformer **IIIb**-3

Center Number	Atomic Number	Atomic Type	Coordinates (Angstroms)		
			X	Y	Z
1	6	0	0.201324	3.162592	0.540205
2	8	0	-0.006165	4.441794	0.997805
3	6	0	-1.059858	5.169103	0.354505
4	1	0	-1.050750	6.165803	0.794905
5	1	0	-2.028962	4.699411	0.535805
6	1	0	-0.878958	5.247001	-0.723395
7	6	0	-0.697784	2.147900	0.920605
8	6	0	-0.450496	0.808998	0.577505
9	6	0	-1.442505	-0.290894	0.927005
10	6	0	-2.508707	-0.464985	-0.157795
11	1	0	-3.107399	0.445920	-0.243295
12	6	0	-3.424617	-1.666377	0.136905
13	8	0	-2.676228	-2.860683	0.266805
14	1	0	-2.898632	-3.402081	-0.505595
15	1	0	-3.955716	-1.477372	1.075005
16	6	0	-4.454019	-1.842168	-0.969895
17	8	0	-5.312910	-0.821461	-1.042795
18	6	0	-6.305311	-0.901252	-2.089095
19	1	0	-6.917618	-1.794147	-1.960395
20	1	0	-5.822311	-0.932356	-3.066195
21	1	0	-6.906803	-0.001947	-1.984695
22	1	0	-1.996908	-0.627489	-1.110495
23	8	0	-2.141503	-0.011788	2.162805
24	6	0	-1.413206	-0.367894	3.338205
25	1	0	-0.444402	0.142197	3.380105
26	1	0	-1.259815	-1.450996	3.376205
27	1	0	-2.019703	-0.060089	4.189605
28	1	0	-0.916614	-1.236498	1.036205
29	6	0	0.692402	0.525188	-0.177995
30	6	0	1.546010	1.534880	-0.647595
31	6	0	1.306222	2.879282	-0.257295
32	6	0	2.219332	4.016575	-0.647295
33	1	0	1.973435	4.388077	-1.644595
34	1	0	3.261729	3.700866	-0.682095
35	1	0	2.111539	4.830475	0.067505

36	8	0	0.882590	-0.806114	-0.524995
37	6	0	2.128385	-1.364525	-0.239595
38	6	0	3.272690	-0.861135	-0.855295
39	8	0	3.214598	0.111766	-1.828295
40	6	0	2.514308	1.294072	-1.752095
41	6	0	2.219076	-2.481325	0.596605
42	6	0	0.997770	-3.099415	1.228105
43	1	0	0.149570	-3.124608	0.543305
44	1	0	0.686675	-2.539012	2.115305
45	1	0	1.210662	-4.121217	1.547105
46	6	0	3.479671	-3.040936	0.823805
47	1	0	3.559263	-3.913937	1.465305
48	6	0	4.627075	-2.506346	0.239005
49	8	0	5.873071	-3.022557	0.448905
50	1	0	5.808565	-3.774956	1.045805
51	6	0	4.523585	-1.411845	-0.614295
52	1	0	5.394888	-1.009553	-1.112695
53	8	0	2.719215	2.085970	-2.630995
54	8	0	-1.782081	2.524909	1.635005
55	1	0	-2.214688	1.709813	1.962905
56	8	0	-4.466227	-2.804368	-1.702595

Conformer **IIIb**-15

Center	Atomic	Atomic	Coordinates (Angstroms)		
Number	Number	Type	X	Y	Z
1	6	0	0.194607	3.159899	0.538705
2	8	0	-0.015990	4.439100	0.994805
3	6	0	-1.072188	5.163002	0.351505
4	1	0	-2.039689	4.690204	0.533105
5	1	0	-0.891588	5.241302	-0.726395
6	1	0	-1.066086	6.159802	0.791905
7	6	0	-0.702895	2.143601	0.918605
8	6	0	-0.452498	0.804901	0.576605
9	6	0	-1.444301	-0.295697	0.924705
10	6	0	-2.510502	-0.467594	-0.160695
11	1	0	-3.107699	0.444407	-0.246195
12	6	0	-3.428604	-1.667592	0.133505
13	8	0	-2.682307	-2.862794	0.263805
14	1	0	-2.902609	-3.403293	-0.509895
15	1	0	-3.959804	-1.477591	1.071305
16	6	0	-4.457905	-1.840790	-0.973895
17	8	0	-5.316402	-0.819488	-1.044695
18	6	0	-6.309103	-0.897085	-2.090795
19	1	0	-6.921705	-1.790084	-1.963395

20	1	0	-5.826503	-0.926786	-3.068095
21	1	0	-6.910300	0.002216	-1.984695
22	1	0	-1.998702	-0.630896	-1.113295
23	8	0	-2.143500	-0.017195	2.160505
24	6	0	-1.417601	-0.378497	3.336205
25	1	0	-0.447400	0.128601	3.380505
26	1	0	-1.267604	-1.462097	3.371605
27	1	0	-2.024401	-0.070896	4.187505
28	1	0	-0.918803	-1.241598	1.032605
29	6	0	0.692901	0.522098	-0.175395
30	6	0	1.545703	1.533496	-0.644595
31	6	0	1.301606	2.878096	-0.256195
32	6	0	2.213009	4.017594	-0.644195
33	1	0	2.100411	4.831595	0.069805
34	1	0	1.969310	4.387995	-1.642495
35	1	0	3.256408	3.704892	-0.675395
36	8	0	0.885698	-0.809103	-0.518495
37	6	0	2.132896	-1.365006	-0.233295
38	6	0	3.272098	-0.862308	-0.850095
39	8	0	3.218400	0.111492	-1.822195
40	6	0	2.518403	1.295394	-1.744995
41	6	0	2.227194	-2.483106	0.607005
42	6	0	1.005492	-3.097303	1.241205
43	1	0	0.686194	-2.522802	2.116405
44	1	0	1.223990	-4.111403	1.579005
45	1	0	0.161492	-3.138801	0.551705
46	6	0	3.483792	-3.042909	0.834005
47	1	0	3.586290	-3.911709	1.472705
48	6	0	4.630394	-2.509912	0.245305
49	8	0	5.822092	-3.111314	0.530705
50	1	0	6.528193	-2.664116	0.053105
51	6	0	4.525296	-1.416811	-0.608795
52	1	0	5.384097	-0.997313	-1.120395
53	8	0	2.730905	2.089193	-2.620595
54	8	0	-1.788594	2.518404	1.631305
55	1	0	-2.218596	1.702305	1.960905
56	8	0	-4.470507	-2.801390	-1.708695

Table S4. Experimental (**1** and **2**) and computed (a and b) ^{13}C-NMR chemical shifts.

Position	**1**	**Ia**	**Ib**	**2**	**Ia**	**Ib**
1	113.6	116.4	116.3	113.7	116.3	116.2
2	156.2	153.7	153.6	156.2	153.8	153.7
3	113.8	114.1	114.0	113.7	114.0	113.9
4	154.2	148.1	147.8	154.2	148.1	147.9
5	144.5	142.1	142.2	144.6	142.1	142.2
6	137.2	130.3	130.5	137.3	130.3	130.4
7	163.8	163.2	163.3	163.7	163.3	163.3
8	75.9	78.9	80.1	75.4	78.7	79.9
9	40.2	40.3	42.3	38.7	40.0	42.0
10	67.3	69.3	68.4	67.4	69.0	68.2
11	175.4	179.0	179.0	175.6	179.1	179.1
12	14.4	16.4	15.6	14.4	16.0	15.3
1′	153.3	153.1	152.9	153.1	153.1	152.9
2′	143.4	138.3	138.4	143.5	138.3	138.4
3′	131.0	134.2	134.4	131.0	134.2	134.4
4′	114.5	116.0	115.9	114.5	116.0	115.8
5′	144.6	157.6	157.5	144.7	157.6	157.6
6′	106.0	101.3	101.0	106.0	101.2	100.9
7′	18.2	19.9	19.3	18.1	19.6	19.0
8-OCH_3	58.1	58.0	58.0	57.9	57.8	57.8
11-OCH_3	60.4	54.2	54.1	60.4	54.0	53.9
5′-OCH_3	52.9	54.8	54.4	52.8	54.5	54.2

Position	**1**	**IIa**	**IIb**	**2**	**IIa**	**IIb**
1	113.6	114.2	113.7	113.7	114.1	113.6
2	156.2	155.3	155.0	156.2	155.3	155.1
3	113.8	114.5	113.0	113.7	114.4	112.9
4	154.2	155.3	155.2	154.2	155.4	155.3
5	144.5	142.9	143.3	144.6	142.9	143.3
6	137.2	139.3	140.9	137.3	139.3	140.9
7	163.8	161.7	161.3	163.7	161.8	161.3
8	75.9	75.3	80.0	75.4	75.1	79.9
9	40.2	40.5	38.5	38.7	40.2	38.2
10	67.3	68.5	71.9	67.4	68.3	71.7
11	175.4	176.7	175.8	175.6	176.8	175.9
12	14.4	16.3	15.5	14.4	15.9	15.1
1′	144.6	150.9	150.6	144.7	150.9	150.6
2′	143.4	136.9	136.7	143.5	136.9	136.7
3′	131.0	133.0	133.2	131.0	133.0	133.2
4′	114.5	112.2	112.1	114.5	112.1	112.0

5′	153.3	153.1	152.8	153.1	153.2	152.9
6′	106.0	105.2	104.2	106.0	105.1	104.1
7′	18.2	19.4	18.9	18.1	19.1	18.6
5-OCH_3	52.9	57.7	57.7	52.8	57.5	57.5
8-OCH_3	58.1	56.7	56.6	57.9	56.5	56.3
11-OCH_3	60.4	53.4	52.4	60.4	53.2	52.1

Position	**1**	**IIIa**	**IIIb**	**2**	**IIIa**	**IIIb**
1	113.8	113.32	113.1	113.7	113.24	113.02
2	156.2	157.69	156.76	156.2	157.73	156.8
3	113.8	112.23	112.74	113.7	112.15	112.66
4	154.2	155.87	155.91	154.2	155.91	155.95
5	144.5	142.66	142.43	144.6	142.66	142.43
6	137.2	141.61	141.34	137.3	141.61	141.34
7	163.8	161.45	161.43	163.7	161.5	161.48
8	75.9	77.36	77.49	75.4	77.18	77.31
9	40.2	44.28	42.09	38.7	44.01	41.81
10	67.3	67.06	67.19	67.4	66.85	66.98
11	175.4	176.48	177.03	175.6	176.57	177.12
12	14.4	15.72	15.79	14.4	15.37	15.44
1′	143.4	142.55	142.79	143.5	142.55	142.8
2′	144.6	144.66	145.19	144.7	144.67	145.2
3′	106.0	102.64	103.67	106.0	102.53	103.56
4′	153.3	152.96	153	153.1	152.99	153.03
5′	114.5	112.26	111.75	114.5	112.17	111.67
6′	131.0	133.17	132.66	131.0	133.15	132.63
7′	18.2	19.5	19.93	18.1	19.16	19.59
5-OCH_3	52.9	57.44	57.56	52.8	57.21	57.32
8-OCH_3	58.1	55.19	56.15	57.9	54.95	55.91
11-OCH_3	60.4	52.99	53.1	60.4	52.74	52.85

Table S5. Experimental (**1** and **2**) and computed (a and b) ^{1}H-NMR chemical shifts.

Position	**1**	**Ia**	**Ib**	**2**	**Ia**	**Ib**
8	5.65	5.58	5.48	5.65	5.55	5.45
9a	1.93	2.43	2.34	2.25	2.48	2.39
9b	2.41	2.43	2.04	2.45	2.47	2.09
10	4.53	4.42	4.60	4.36	4.41	4.59
12	2.38	2.37	2.47	2.39	2.41	2.51
4′	6.47	6.64	6.59	6.47	6.59	6.53
6′	6.65	6.67	6.61	6.63	6.61	6.54
7′	2.46	2.31	2.42	2.47	2.35	2.46
8-OCH_3	3.54	3.28	3.53	3.40	3.31	3.55

11-OCH_3	3.79	3.75	3.76	3.79	3.76	3.77
5′-OCH_3	3.78	3.71	3.76	3.81	3.72	3.78
4-OH	9.07	9.17	9.09	9.27	9.34	9.28

Position	**1**	**IIa**	**IIb**	**2**	**IIa**	**IIb**
8	5.65	5.51	5.53	5.65	5.48	5.49
9a	1.93	2.15	2.01	2.25	2.20	2.08
9b	2.41	2.60	2.53	2.45	2.65	2.58
10	4.53	4.39	4.43	4.36	4.39	4.43
12	2.38	2.43	2.43	2.39	2.48	2.48
4′	6.47	6.39	6.57	6.47	6.34	6.51
6′	6.65	6.94	6.67	6.63	6.87	6.60
7′	2.46	2.35	2.28	2.47	2.40	2.34
5-OCH_3	3.78	3.78	3.83	3.81	3.79	3.84
8-OCH_3	3.54	3.29	3.48	3.40	3.32	3.50
11-OCH_3	3.79	3.75	3.83	3.79	3.76	3.84
4-OH	9.07	9.17	9.09	9.27	9.34	9.28

Position	II	3a	3b	I	3a	3b
8	5.65	5.66	5.74	5.65	5.61	5.68
9a	1.93	2.18	1.78	2.25	2.24	1.87
9b	2.41	2.15	2.27	2.45	2.21	2.33
10	4.53	4.35	4.63	4.36	4.34	4.61
12	2.38	2.46	2.48	2.39	2.51	2.53
3′	6.65	6.41	6.46	6.63	6.34	6.38
5′	6.47	6.66	6.47	6.47	6.57	6.39
7′	2.46	2.64	2.62	2.47	2.69	2.68
5-OCH_3	3.78	3.84	3.84	3.81	3.85	3.85
8-OCH_3	3.54	3.46	3.55	3.40	3.48	3.57
11-OCH_3	3.79	3.71	3.73	3.79	3.72	3.75
4-OH	9.27	9.34	9.28	9.07	9.17	9.09

Table S6. Statistics of Ordinary Least Squares Linear Regression (OLS-LR) of experimental and computed ^{13}C- and ^{1}H-NMR chemical shifts.

Type	Experimental	Compound	CMAD[a]	CLAD[b]	R^2	*RMSE*	*F*	*p* value
C	**2**	**Ia**	3.2	12.9	0.9920	4.6	2482.05	< 0.01
C	**2**	**Ib**	3.2	12.9	0.9917	4.6	2399.26	< 0.01
C	**2**	**IIa**	2.1	7.2	0.9963	3.1	5439.24	< 0.01
C	**2**	**IIb**	2.5	8.3	0.9951	3.6	4095.03	< 0.01
C	**2**	**IIIa**	**2.2**	**7.7**	**0.9965**	3.0	5744.66	< 0.01
C	**2**	**IIIb**	**2.0**	**7.6**	**0.9971**	2.7	6931.61	< 0.01
C	**1**	**Ia**	3.2	13.0	0.9921	4.5	2497.03	< 0.01

C	**1**	**Ib**	3.2	12.9	0.9919	4.6	2454.72	<0.01
C	**1**	**IIa**	2.1	7.0	0.9964	3.1	5462.78	<0.01
C	**1**	**IIb**	2.6	8.0	0.9951	3.6	4061.24	<0.01
C	**1**	**IIIa**	**2.1**	**7.4**	**0.9968**	2.9	6199.11	<0.01
C	**2**	**IIIb**	**2.0**	**7.3**	**0.9973**	2.7	7277.87	<0.01
H	**2**	**Ia**	0.08	0.23	0.9959	0.11	2162.44	<0.01
H	**2**	**Ib**	0.13	0.36	0.9892	0.18	821.91	<0.01
H	**2**	**IIa**	0.10	0.24	0.9938	0.14	1432.35	<0.01
H	**2**	**IIb**	0.09	0.17	0.9956	0.11	2051.67	<0.01
H	**2**	**IIIa**	**0.11**	**0.29**	**0.9952**	0.16	2079.63	<0.01
H	**2**	**IIIb**	**0.15**	**0.38**	**0.9923**	0.20	1283.78	<0.01
H	**1**	**Ia**	0.13	0.50	0.9859	0.21	629.48	<0.01
H	**1**	**Ib**	0.13	0.41	0.9869	0.20	675.50	<0.01
H	**1**	**IIa**	0.14	0.29	0.9898	0.18	869.99	<0.01
H	**1**	**IIb**	0.08	0.18	0.9966	0.10	2599.94	<0.01
H	**1**	**IIIa**	**0.14**	**0.26**	**0.9942**	0.18	1708.42	<0.01
H	**1**	**IIIb**	**0.09**	**0.19**	**0.9975**	0.12	3932.50	<0.01

[a] CMAD = corrected mean absolute deviation, computed as $(1/n)\sum_{i}^{n} |\delta_{\mathrm{calc}} - \delta_{\mathrm{exp}}|$, where δ_{calc} and δ_{exp} refer to the calculated and experimental chemical shifts. [b] CLAD = corrected largest absolute deviation, computed as $\max(|\delta_{\mathrm{calc}} - \delta_{\mathrm{exp}}|)$.
